# Affiliation in human-AI interactions is based on shared psychological traits

**DOI:** 10.1038/s44271-026-00433-8

**Published:** 2026-03-23

**Authors:** Santiago Castiello, Riddhi Jain Pitliya, Daniel R. Lametti, Robin A. Murphy

**Affiliations:** 1 Wu Tsai Institute, Yale University, New Haven, USA; 2 Department of Experimental Psychology, University of Oxford, Oxford, UK; 3 Department of Psychology, Acadia University, Wolfville, Canada; 4 OneReach.AI, Denver, USA

**Keywords:** Human behaviour, Social behaviour

## Abstract

People affiliate with others who share their psychological traits. Does the same phenomenon occur with AI instructed to mimic human psychology? Large language models (LLM) were prompted to use language that mimicked anxious symptoms or their absence (Experiment 1; n = 100), extroversion or introversion (Experiment 2; n = 100), and an exact mirror or inverse of participants’ personality (preregistered Experiment 3; n = 100). With full knowledge that they were interacting with an artificial system, participants engaged in written interactions with both LLM versions and then evaluated their engagement. Those with anxiety reported a stronger connection to the LLM that mimicked anxiety, a distinction also reflected in the sentiment of the messages they exchanged. Extroverted participants affiliated more with the AI that mimicked extroversion. Finally, when participants interacted with LLMs that mimicked either their own personality profile or the inverse of their personality (i.e., the opposite pattern of their Big-Five scores), they reported more affiliation with the LLM mimicking their personality; this distinction was also reflected in the sentiment of their messages. Results support affiliation in human-AI interactions based on the linguistic presentation of a shared psychology. We propose that through *socioaffective* tuning, LLMs might achieve greater human-like correspondence.

## Abstract (web summary)

Birds of a feather flock together: Despite full knowledge that they’re chatting with an AI, humans affiliate more with LLMs that, through language, mimic their own personality traits like anxiety and extroversion.

## Introduction

Humans tend to affiliate more with people who share their likes, interests, and even mental states^1,2^. Is this affiliative tendency—or something like it—possible between artificial intelligence (AI) and humans? As language facilitates social interaction, the emergence of Large Language Models (LLMs) has expanded the range of groups we might interact with to include artificial systems. LLMs readily adopt specific linguistic styles in response to prompts; we can now create person-centered or bespoke AI with the aim of building stronger human-AI bonds. But this idea—long a plot point for science fiction writers (see, for instance, the 2013 Spike Jonze film *Her*)—has little empirical support, largely because AI with the ability to mimic human-*like* language skills are relatively new. Here, we use LLMs to provide evidence of a type of socioaffective alignment^3^ with AI. We prompted the LLMs GPT-4 and GPT 4.1 to produce language associated with mental health states (i.e., anxious vs non-anxious), personality traits (i.e., extroverted vs introverted), or a personality profile that mirrored participant personalities to test whether human-AI affiliation based on a shared psychology might occur.

In human interactions, people tend to affiliate with other individuals who share characteristics with themselves^1,4^. For example, people choose partners who resemble themselves, a phenomenon observed in both in-person and app-based dating^5,6^. During social interactions, this affiliative tendency is reflected in shared patterns of neural activity between friends^7^, and individuals assuming beliefs of their close network^8^. Factors that contribute to this effect include things like shared religious beliefs, interests, hobbies, personality characteristics, mental states, and social network distance^1^. In short, humans tend to like other humans who are a reflection of aspects of themselves. As language-based AI companions increasingly become a part of our lives and do a better job of mimicking human traits^9^, it is important to investigate whether humans might also prefer AI that reflect aspects of themselves.

There is evidence that mimicry of a ‘psychology’ in artificial systems might matter to humans. Meta-analytical evidence supports the idea that participants’ personality matters for various forms of acceptance of robots^10^. In situations where people are able to identify dominant or submissive language in pre-programmed computer responses they feel more attracted to computers that are more similar to themselves in these traits^11^. A similarity-attraction effect is also found when people hear computer-synthesized speech^12^. And, more recently, extroverted participants were shown to deem scripted interactions with consumer-oriented chatbots as more enjoyable when the bot responded more quickly and used extroverted language and punctuation (e.g., “Hi there!” instead of “Hello”)^13^. All the above studies broadly support the idea that affiliation, connection, and attraction can emerge during interactions with artificial agents. LLMs present an AI-based tool to systematically test the limits of these effects.

LLMs, which generate contextually correct, human-like language in real time, have the ability to mimic human personalities in the language they convey^14^. The aim of the current experiments was to test whether, in a short unconstrained conversation, humans might affiliate more with an LLM that, through language, exhibits a shared psychological trait, despite full knowledge that they are talking to a computer program. Alternatively, LLMs that are too *human*-like in the personality they convey may drive feelings of unease—an effect similar to the “uncanny valley” phenomenon noted in some human-robot interactions^15^.

**Fig. 1:**
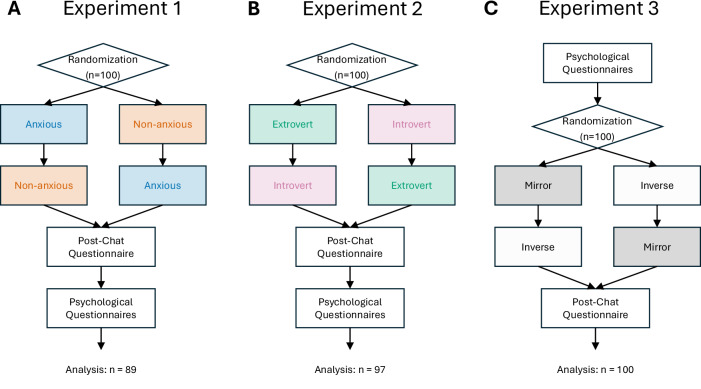
Randomized cross-over Controlled Experimental designs. **A**–**C** Diagram depicting the flow of tasks for Experiments 1, 2, and 3. Each coloured box corresponds to a condition consisting of a free conversation with an LMM prompted to mimic a personality trait or profile. Thus, two conditions per experiment. The order of the condition presentation was randomized before participants started the experiment. Post-Chat questionnaire involved questions to measure affiliation, the main dependent variable. The Psychological Questionnaires boxes in (**A**, **B**) were used to measure anxiety and extraversion, respectively; in (**C**) it was used to prompt the LLMs to reflect a specific personality profile as captured by the Mirror and Inverse conditions (see Methods).

In Experiment 1, the LLM GPT-4 was prompted using clinically relevant criteria—specifically, answers to the twenty-item State Trait Anxiety Inventory reflecting an anxious psychological state e.g., “I almost always feel nervous and restless” ^16^—to express through language a psychology aligned with either an anxious or non-anxious psychological state. Participants conversed with both versions of GPT-4 and then answered a series of questions designed to test the extent to which they affiliated—expressed feelings of similarity or distance—with the AI (Fig. 1A). Participants’ own anxiety was then measured, and we examined their affiliation with each GPT-4 persona in relation to their reported anxiety levels. We hypothesized that participants’ anxiety scores would predict affiliation towards the LLM that produced anxious language.

In Experiment 2, we examined whether the effects observed in Experiment 1 generalized to personality traits. GPT-4 was prompted using answers to twelve questions from the International Personality Item Pool to produce language aligned with either an extroverted or introverted personality^17^ (Fig. 1B). Following conversations with each version of the LLM, participants’ affiliation to the AI was measured in relation to reported extroversion. We hypothesised that extroverted participants would affiliate more with an LLM that used extroverted language and vice versa for introverted participants.

In a final *preregistered* experiment, we first measured participants’ personality and then, for each participant, we created bespoke versions of the LLM (GPT 4.1) that mirrored the language aligned with either their exact personality profile or the inverse of their personality profile (i.e., an LLM that displayed the exact opposite pattern of Big 5 personality scores in the language it used). Following conversations with each version of the LLM we measured their affiliation with the AI (Fig. 1C). As in Experiments 1 and 2, we predicted that participants would affiliate more with the LLM that used language that mirrored their psychology. Finally, in each experiment, we analysed the sentiment of the messages participants sent to the LLMs they conversed with. The goal of this analysis was to examine if the tone of the language they used in conversation with each AI reflected their self-reported affiliative tendencies.

## Methods

### Participants

We recruited 100 participants via Amazon Mechanical Turk (MTurk) for Experiment 1. As the effects in Experiment 1 were large, we used the same sample size for Experiment 2. Fourteen participants in total were excluded from the final analysis for failing to engage with the LLM enough (see *Language Model Design*) making the final sample size 89 for Experiment 1 and 97 for Experiment 2. Experiment 1 consisted of 35 female participants, 53 male participants, and 1 nonbinary, with a mean (M) age of 39.6, standard deviation (SD) of 7.45, and ranging from 18 to 50. Experiment 2 consisted of 49 female participants and 47 male participants, with a M of 42.11, SD of 6.11, and range between 25 to 50. Experiment 3 was preregistered (23/06/2025 https://aspredicted.org/9yg6-y3xp.pdf) and conducted in Prolific. Experiments 1 and 2 were not preregistered.

We calculated the sample size with the function *pwr.t.test*, which was based on a previous pilot; we required 90 participants to detect an effect size of 0.3 with a power of 0.8 and an alpha of 0.05, and thus we recruited 100 participants. Fifty-seven participants were male, 42 were female, and 1 was nonbinary. Gender was determined by participants self-identification: “What is your gender?” The mean age was 34.86 (SD 8.06) and ranged between 19 and 50. All participants were native English speakers; they were paid $7 (USD) for completing the experiment, which took approximately 30 min. The University of Oxford’s Central University Research Ethics Committee (CUREC: R86261/RE001) approved the study.

### Software and materials

The LLM interactions were designed using the conversational AI platform Generative Studio X (GSX, OneReach.ai). In Experiments 1 and 2, the conversations were embedded in the Gorilla Experiment Builder (www.gorilla.sc)^18^. Experiment 3 was run entirely within GSX. Statistical analyses and visualizations were conducted in R studio^19^ with the R packages *gtools, reshape2, ggplot2, ggpubr, dplyr, Hmisc, report, lme4, lmerTest, pwr, tidytext, DescTools, and effectsize*.

#### Language model design

Participants had two text-based conversations with different versions of the large language model GPT-4. For Experiments 1 and 2, the latest version of GPT-4 was used at the time of testing—October 2023 for Experiment 1 and August 2024 for Experiment 2. Experiment 3, conducted in June 2025, used GPT-4.1. Regardless of the version, GPT was instructed via the LLM’s system message to use language like a person chatting with a friend. In Experiment 1, the LLM was instructed to mimic either an anxious or non-anxious person and in Experiment 2 extroverted or introverted traits. Prompts also included answers to the twenty item State-Trait Anxiety Inventory (STAI^16^) to reflect either an anxious or non-anxious state (Experiment 1), and to the twelve questions from the International Personality Item Pool^17^ to reflect either an extroverted or introverted personality (Experiment 2); also, (i) instructions to never reveal the LLM’s identity, but to show interest in the conversational partner, and keep responses to 2 or 3 sentences, (ii) the LLM’s name (either Pat or Alex; counterbalanced), and (iii) two conversational turns as example responses. See an example of the chat here: https://chat.staging.onereach.ai/p91GBglaSBSeIFOOdGiKgA/05i2cuj.

For Experiment 3, the AI was instructed to adopt a personality profile in its language and responses described by the participant’s scores (or the inverse of their scores) from the Big Five Inventory with 44 items (BF-I44). The prompt included examples of language associated with each personality trait. See section “LLM Prompts” within Supplementary Information for all the prompts used in the study.

When chatting with participants, in Experiments 1 and 2 the LLM’s context window was restricted to eight conversational turns. At this point, the first two turns were ejected after every subsequent turn so that the context window never grew beyond the prompt, example responses, and the most recent eight turns. Limiting the LLM’s memory to the prompt and the last eight turns ensured that the model’s persona was always a strong reflection of the prompt and the most recent exchanges with participants, and did not drift towards language used by the participant. In Experiment 3, the LLM’s context window was not limited (i.e., the LLM had access to the entire conversation). This change was made because, during pilot testing, GPT 4.1 was found to be significantly better at following instructions than GPT 4.

In Experiments 1 and 2, each chat ended after 31 conversational turns or 10 min—whichever came first. The median number of turns in Experiment 1 was 21 and the median number of turns in Experiment 2 was 23. Participants who completed fewer than 8 turns—or more than 2 standard deviations away from the mean number of turns—were excluded. Fewer than eight turns were also selected as an exclusion criterion in these experiments because it was the length of the LLM’s context window. In Experiment 3 participants were required to complete 24 conversational turns regardless of how long the conversations took.

#### Questionnaires

In addition to the post-chat Likert questions—where we asked participants their impressions about their chats with Pat and Alex (see section “Post chat questionnaire” within the Supplementary Information)—participants in Experiment 1 completed the ninety-item Symptom Checklist Revised (SCL-90^20^); they also completed the ten-item version of the Big Five Inventory (BFI-10^21^). Participants in Experiment 2 completed the forty-four item version of the BFI-44^22^. Participants in Experiment 3 also completed the BFI-44 before their interactions with the LLMs. All questionnaires’ scores were normalized between 0 and 1.

### Procedure

In Experiments 1 and 2, after completing the consent form, participants were informed that they would be interacting with two AI ‘chatbots’ via text-based conversations. The goal of these conversations was to determine if they would get along with the AI if it were a real person. The AI always started the conversations. They were told the name of the AI they would be chatting with and then the chat began. When the conversation ended, they were introduced to the second AI and they began that conversation. The names of the AI and the psychological personas they mimicked were counterbalanced across participants in each experiment. Following the chats, participants completed a series of questionnaires delivered in a fixed order. First, they completed the post-chat questionnaire to assess how similar or different they felt to each AI (i.e., affiliation); then they completed the psychological questionnaires to measure anxiety (Experiment 1) or introversion-extroversion (Experiment 2). There was no effect of chatbot order on affiliation (see section “Order effect” within Supplementary Information).

The procedure for Experiment 3 was identical to Experiments 1 and 2 with the exception that participants completed the psychological questionnaire *before* their interactions with the LLMs. The order of tasks in the experiments is depicted in Fig. 1.

### Data analysis

The experiments used a bespoke post-chat questionnaire (see “Post chat questionnaire” in Supplementary Information). The questions captured a specific aspect of the participant’s affiliation experience:

1. “I felt that we are similar” = “similar”
2. “I enjoyed our conversation” = “enjoy”
3. “I would chat with them again” = “chat-again”
4. “I felt that they were different from me” = “different” *
5. “I felt distant from them” = “distant” *
6. “I felt that they understood me” = “understood”

Participants rated each statement using a five item Likert scale that ranged from “Strongly Disagree” (coded as −2) to “Strongly Agree” (coded as +2). Items with * (different and distant) were inversely coded, thus disagree is evidence for affiliation. To capture participants’ overall affiliation towards each version of the LLM, we took the average across questions for each participant resulting in a single affiliation score per participant and per chat. The internal consistency of the bespoke questionnaire was measured with Cronbach’s alpha, which was greater than 0.88 for all three experiments. One factor was the best latent model structure of the six items (Supplementary Fig. S1). Our affiliation score was highly correlated [r(49) = 0.89 (95% CI from 0.82 to 0.94), R^2^ = 0.79, *p* < 0.001] with the Connection During Conversations Scale (CDCS^23^). See section “Affiliation Score” within Supplementary Information for consistency, factor structure, and validation of the questionnaire.

Anonymized data associated with the consent form and post chat questions were handled by Gorilla Experiment Builder (www.gorilla.sc) (Experiments 1 and 2)^18^ or GSX (Experiment 3). Using participants’ questionnaire responses to the SCL-90 (Experiment 1) and the BFI-44 (Experiment 2), we calculated an anxiety score for participants in Experiment 1 and an extroversion score for participants in Experiment 2, respectively.

### Statistical analysis

For experiments 1 and 2, we planned to fit a Linear Mixed Model predicting affiliation score (${AS}$) with LLM type, questionnaire, and their interaction:

1 $${AS} \sim {\beta }_{0}+{\beta }_{1}* {questionnaire}+{\beta }_{2}* {LLM}\,\\ {type}+{\beta }_{3}* {questionnaire}* {LLM\_type}$$

Then if the interaction ${\beta }_{3}$ was significant, we planned to fit two simple linear models, one for each LLM type:

2 $${AS} \sim {\beta }_{0}+{\beta }_{1}* {questionnaire}$$

However, we decided to use a non-parametric approach for three reasons. First, if we used ${AS}$ as the outcome, only two observations per participants make unsuitable the use of Linear Mixed Models; second, if we use all the six individual questions the residuals were not normally distributed (one-sample Kolmogorov-Smirnov test: Expt 1, D = 0.068, *p* < 0.001; Expt 2, D = 0.074, *p* < 0.001). Therefore, the non-parametric approach followed the two-step logic—first estimating the interaction (correlating affiliation score difference between conditions against the questionnaire), then the effects for each condition. We present the parametric analysis in the section “Sensitivity Analysis: Main Results with Parametric Statistics” within Supplementary Information. The results point to the same direction.

We estimated the effect of the interaction by correlating the questionnaire scores (anxiety for Expt. 1 and extroversion for Expt. 2) against the ${AS}$ difference between LLM types (Expt. 1: Anxious-Non-anxious, and Expt. 2 Extrovert-Introvert). If this correlation was significant, we then pursued two additional correlations, one for each condition. We used two-sided Spearman rank correlation coefficient (ρ) because neither anxiety (Expt 1) nor extraversion (Expt 2) were normally distributed (Expt 1, D = 0.27, *p* < 0.001, Expt 2, D = 0.14, *p* < 0.001).

For Experiment 3, the main hypothesis was tested with a pairwise *t-test* between the two conditions. This analysis was preregistered on the 23rd of June 2025 (https://aspredicted.org/9yg6-y3xp.pdf). As anticipated before, AS was not normally distributed (one-sample Kolmogorov-Smirnov test: D = 0.13, *p* = 0.003), thus we also tested this hypothesis with a Wilcoxon test (both results corroborated the hypothesis).

We also preregistered that participants’ affiliation score would be moderated by how different the personalities between the Mirror and Inverse LLM personalities were (put simply, participants would affiliate more with the LLM that mirrored their personality if their personality and inverse personality were very different); this was tested with an interaction as in Eq. 1. We called this measure, condition distance, and it was calculated by the sum of the squared root difference between the vectors containing the personality scores for Mirror, **m**, and Inverse, **i** (bold denotes a vector) personalities. Therefore:

3 $${condition}\,{distance}= {\sum}_{i=1}^{5}{({m}_{i}-{i}_{i})}^{2},$$

Usually, Likert items are scored between 1 and 5, in our case we subtracted 3 so they ranged between -2 (Disagree strongly) to +2 (Agree strongly). Then we calculated the personality scores by averaging the items for each dimension (see section “Big-Five-44 items” in the Supplementary Information to see the exact items used for each personality). The inverse personality condition was calculated by multiplying the Mirror personality vector **m** by −1 (**i** = −1 * **m**). For example, a participant with a personality of Openness = 2, Conscientiousness=1, Extraversion=0, Agreeableness = −1, and Neuroticism = −2], then, **m** = [2,1,0,-1,−2] and **i** = [−2,−1,0,1,2].

The statistical threshold, $\alpha$, for all tests was 0.05. The Holm-Bonferroni method was used to correct for multiple comparisons across the six individual outcomes questions in the post-chat questionnaire (Supplementary Fig. S2). We report 95% confidence intervals for effect sizes.

### Sentiment Analysis

In addition to the relationship between participants’ affiliation scores and their psychological traits, we also examined the sentiment of the messages sent by both participants and the different LLM personas used in the study. We did this to assess the extent to which the sentiment or tone of participants messaged might come to reflect the sentiment of the LLM they were chatting with—an implicit marker of affiliation.

The sentiment of messages was assessed by another version of GPT-4 using the following prompt as the LLM’s system message: *Analyze the sentiment of messages. Given a message, classify it as positive, negative, neutral, or mixed. Return just the sentiment of the message. Do not return anything else. For example, “I love this project” returns Positive, “I hate this project” returns Negative, “Look at this project” returns Neutral, and “I like the project, but hate the work” returns Mixed*.

For each participant and LLM, the number of messages with a positive, negative, neutral, and mixed sentiment were found for both the participant and the LLM. To control for differences in chat length between participants, the number of messages in each sentiment category were divided by the total number of messages sent by each participant or LLM. This gave a normalized measure of sentiment for each sentiment category that was then compared between LLM types in each experiment. Differences in the sentiment of messages between LLM types was examined using two-factor ANOVAs—4 (sentiment) x 2 (LLM type); we then explored significant interactions with paired t-tests and reported Cohen’s d as effect sizes.

In Experiments 1 and 2, for each sentiment category, we also examined the *difference* in normalized sentiment between each GPT-4 persona (Experiment 1: anxious *minus* non-anxious; Experiment 2: extroverted *minus* introverted) versus participants’ self-reported psychological traits (anxiety in Experiment 1 and introversion-extroversion in Experiment 2). This relationship was modelled using linear regression, and the slopes of the best fit lines were compared to zero.

### Lexical analysis

To verify that GPT-4 was using language consistent with the prompted psychology trait in Experiments 1 and 2, we used a ‘bag-of-words model’ to identify the top 12 words produced by each version of the LLM across all conversations, removing filler words. To identify words uniquely produced by each LLM version, we also calculated the weighted log-odds ratio between the two LLM personas for all words produced in Experiments 1 and 2^24,25^. This method identified the distinctiveness of words produced by a particular LLM in comparison to the other LLM in the experiment, while accounting for the total amount of text. The resulting weighted log-odds ratio are standardized z-scores; a value greater than 1.96 indicates that a word is significantly more characteristic of one LLM persona than the other at *p* < 0.05. See Supplementary Figs. S3 and S4.

### Open Practices Statement

The R scripts and data used for this work can be found in https://github.com/santiagocdo/chatPersonalities.

## Results

### Experiment 1

#### Anxious participants affiliated more with an LLM that mimicked anxiety

**Fig. 2:**
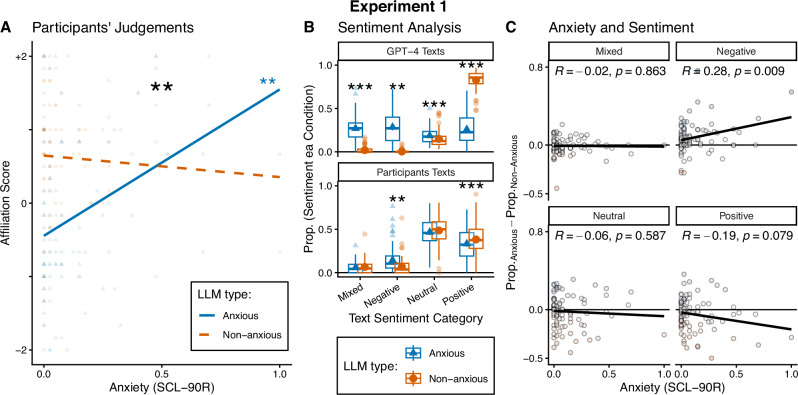
Anxious people affiliate more with LLM mimicking anxiety. **A** Affiliation score as a function of anxiety scores for both LLM personas (Anxious and Non-anxious) as indicated by the colours (*n* = 178; 89 for each condition). **B** Boxplots and average sentiment from the proportion of text categories (Mixed, Negative, Neutral, and Positive) within each condition (LLM type), for both GPT-4 (top panel) and Participants (bottom panel; n[proportions] = 1424; each boxplot 89). Boxplots represent the median, interquartile (IQR) range and the whiskers represent 1.5 the IQR. No displaying statistical significance for GPT-4 Texts because differences are by design. **C** Proportions differences between sentiments used by participants in Anxious versus Non-anxious LLM conditions as a function of participant anxiety score. Each sub-panel represents a sentiment category; the Pearson correlation with its associated p-value is displayed at the top (for each panel, *n* = 89). ***: *p* < 0.001, **: *p* < 0.01, *: *p* < 0.05. Black and big asterisks represent the interaction (difference between slopes), and coloured and small asterisks represent individual slopes against the null hypothesis of 0.

Figure 2a shows how participants’ anxiety was related to their affiliation with each version of the AI. The solid lines are the line of best fit to the data for conversations with either the version of GPT-4 that mimicked an anxious state (blue lines) or non-anxious state (dashed red lines). Overall, participants affiliated more with the AI in the Nonanxious condition (*M* = 0.61) than the Anxious condition (M = −20; t(88) = 5.86, *p* < 0.001, *d* = −0.62 [−0.8 to −0.39]). Participants’ affiliation score was predicted by the extent to which their anxiety score mirrored each LLM persona (Fig. 2A). Specifically, an interaction was observed between their self-reported anxiety and LLM type—anxious or non-anxious [ρ(87) = 0.22, *p* = 0.009, Cohen’s d = 0.57 (95% CI from 0.144 to 1.03)]. Participants with higher anxiety affiliated more with the version of GPT-4 that mimicked anxiety [ρ(87) = 0.30, *p* = 0.004, d = 0.63 (0.2, 1.09)], and that was not the case for the non-anxious condition [ρ(87) = −0.10, *p* = 0.341, *d* = −0.21 (−0.64, 0.22)]. An analysis of participants’ answers to each of the six questions in the post chat questions suggests that this affiliative tendency related to feelings of *similarity* and *understanding* in the case of anxious participants interacting with an anxious GPT-4 (Supplementary Fig. S2); these same participants *disagreed* with feelings of difference and distance towards the anxious version of GPT-4. Taken together, the results provided evidence that, in a short interaction, humans could perceive differences in LLMs based on a human psychology trait the LLM was prompted to mimic, and humans affiliated with LLMs based on these differences.

#### The sentiment of GPT-4’s messages was associated with the sentiment of participant messages

The sentiment of messages sent by both GPT-4 personas and participants was categorized as either Mixed, Negative, Neutral, or Positive (see Methods). This analysis had two aims: 1) to verify that an LLM instructed to mimic a negative emotional state (anxiety) produced more negative messages than an LLM instructed to mimic a positive emotional state (non-anxious); and 2) to test whether the sentiment of participants’ messages was associated with the sentiment of messages sent by the LLM in a manner that might relate to their self-reported affiliation with each LLM version.

As observed in Fig. 2B (GPT-4 Texts), the two LLMs produced messages with markedly different sentiment profiles. To test this, we used a two-factor ANOVA [4 (sentiment) x 2 (LLM type)] and post-hoc paired t-test for each sentiment category. The interaction revealed differences in the GPT-4 personas between the sentiment categories [F(704,3) = 498.64, *p* < 0.001, ${\eta }_{p}^{2}=0.68$ (0.65, 1)]. When GPT-4 was instructed to mimic an anxious state it produced more negative [t(88) = 14.88, *p* < 0.001, *d* = 1.58 (1.26, 1.89)] messages and fewer positive [t(88) = −27.67, *p* < 0.001, *d* = −2.93 (−3.41, −2.45)] messages than when it was instructed to mimic a non-anxious state. To further verify that the LLMs used language that reflected the instructed personality, we identified words that were significantly more likely to be used by each LLM persona (as captured by a weighted log odds ratio, see Supplementary Information). When GPT-4 was instructed to mimic anxiety it uniquely used words like “worry”, “anxious”, and “nervous”; but when it was instructed to mimic a lack of anxiety it uniquely used words like “spirit”, “absolutely”, and “awesome”. Taken together, this suggests that the LLMs mimicked the desired personality trait.

We also ran a sentiment analysis for participants’ texts (Fig. 2B) and found an interaction between the sentiment of the messages they sent and the GPT-4 persona they interacted with [F(704,3) = 7.13, *p* < 0.001, ${\eta }_{p}^{2}=0.03$ (0.01, 1)]. Based on this result, we conducted paired t-tests for each sentiment category. Regardless of their anxiety, participants wrote more positive messages [t(88) = −2.76, *p* = 0.007, *d* = −0.29 (−0.51, −0.08)] and fewer negative messages [t(88) = 4.71, *p* < 0.001, *d* = 0.50 (0.28, 0.72)] when they interacted with the non-anxious GPT-4 persona compared to the sentiment of their messages when they interacted with the anxious GPT-4 persona.

For each sentiment category, we then examined the difference in sentiment between the two versions of GPT-4 versus participants’ self-reported anxiety. As shown in the top right panel of Fig. 2C, anxiety scores positively correlated with a higher proportion of *negative texts* when interacting with the version of GPT-4 instructed to mimic anxiety [*β* = 0.24, *p* = 0.009, Std. Coef.= 0.28 (0.07, 0.48)]. No other significant relationships were observed using this measure.

Taken together, the results in Fig. 2B, C provide evidence that participants tended to mirror the sentiment of GPT-4’s messages, and this phenomenon was associated with their own self-reported anxiety. Thus, in addition to participants’ self-reported affiliation with each version of GPT-4, their linguistic behaviour—specifically, the tone of the language they used in conversation with each LLM—reflected the presence of a shared psychological trait.

### Experiment 2

#### Extroverted participants affiliate more with an LLM that mimicked extroversion

In Experiment 1, we found evidence for affiliation with an AI that mimicked a shared mental health trait—in this case, anxiety. The aim of Experiment 2 was to replicate and extend Experiment 1 to the personality traits of extroversion and introversion. It is possible that using a mental health trait like anxiety to prompt the LLM was more unusual or distinctive, and therefore that the affiliation effect is only found with these mental health-related states. However, if affiliation with LLMs is a general phenomenon reflecting the perception of a shared psychological state—as it can be in human relationships—then we might expect a similar relationship between human personality traits and LLMs instructed to mimic these traits.

Participants engaged in conversations with two versions of GPT-4—one prompted to convey extroversion and the second prompted to convey introversion. As in Experiment 1, participants knew they were chatting with an AI. Following each interaction, participants completed the same post chat questions used in Experiment 1 to assess affiliation with each LLM, and the 44-item Big Five Inventory^22^ (BFI) to assess extroversion-introversion.

**Fig. 3:**
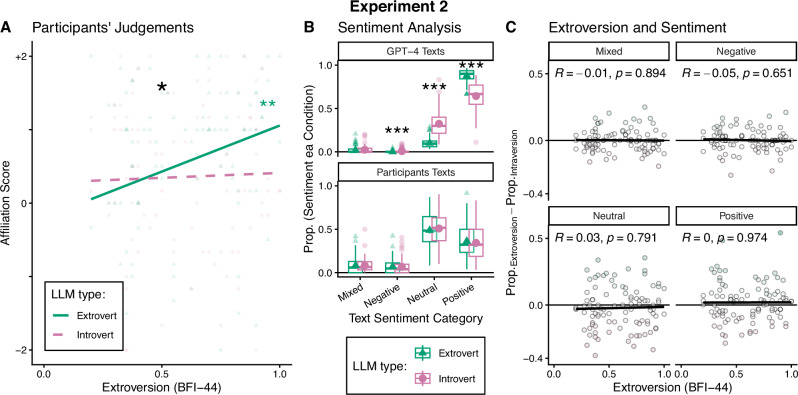
Extroverted people affiliate more with LLM mimicking extraversion. **A** Affiliation score as a function of extroversion scores for both GPT-4 personas (Extrovert and Introvert) as indicated by the colours (*n* = 194; 97 for each condition). **B** Boxplots and average from the proportion of text sentiment categories (Mixed, Negative, Neutral, and Positive) within each condition (GPT-4 type), for both GPT-4 Texts (top panel) and Participant Texts (bottom panel; n[proportions] = 1552; each boxplot 97). Boxplots represent the median, interquartile (IQR) range, and the whiskers are 1.5 the IQR. **C** Proportion differences between participants’ sentiments used in Extrovert versus Introvert conditions as a function of extroversion score. Each sub-panel represents a sentiment category; the Pearson correlation with its associated p-value is displayed at the top (for each panel, *n* = 97). ***p < 0.001, **p < 0.01, *p < 0.05. Black and big asterisks represent the interaction (difference between slopes), and coloured and small asterisks represent individual slopes against the null hypothesis of 0.

Figure 3A shows affiliation with each version of GPT-4 in Experiment 2 as a function of participants’ extraversion-introversion. The solid lines represent the line of best fit to the data for each persona GPT-4 was prompted to mimic, extrovert (green line) and introvert (dashed pink line). Overall, there was **no statistically significant difference** in participants’ affiliation between the Extrovert (*M* = 0.55) and Introvert (*M* = 0.36) conditions (t(96) = 1.43, *p* = 0.157, *d* = 0.14 [-0.06 to.34]). As in Experiment 1, participants’ affiliation score was predicted by whether they shared the psychology trait the LLM was prompted to mimic. Specifically, an interaction was observed between their self-reported extroversion introversion and LLM-type—mimicry of extroversion or introversion [ρ(95) = 0.21, *p* = 0.035, *d* = 0.44 (0.03, 0.87)]. Extroverted participants reported greater affiliation with the version of GPT-4 instructed to mimic extroversion [ρ(95) = 0.28, *p* = 0.006, *d* = 0.58 (0.17, 1.01)]. This was not observed when they interacted with GPT-4 instructed to mimic introversion [ρ(95) = 0.02, *p* = 0.834, *d* = 0.04 (−0.36, 0.45)].

An analysis of participants’ answers to each of the six post chat questions suggests that this affiliative tendency related to feelings of similarity (Supplementary Fig. S2), in the case of extroverted participants interacting with an extroverted GPT-4, and feelings of difference when these same participants interacted with the introverted version of GPT-4. As in Experiment 1, the results provide evidence that humans perceive differences in LLMs based on a human psychology trait the LLMs are prompted to mimic, and we affiliate with LLMs based on these perceived differences.

#### No significant effect between the sentiment of participants’ messages and GPT-4 type

Like Experiment 1, the two GPT-4 personas produced messages with distinct sentiment patterns, reflecting the personality trait the AI were instructed to mimic [two-factor ANOVA, interaction between sentiment and LLM type: F(768,3) = 241.59, *p* < 0.001, ${\eta }_{p}^{2}=0.49$ (0.45, 1)]. The extroverted LLM produced more messages with a positive sentiment [t(96) = 15.81, *p* < 0.001, *d* = 1.61 (1.30, 1.91)], fewer messages with a neutral sentiment [t(96) = −16.01, *p* < 0.001, *d* = −1.63 (−1.93, −1.32)], and less negative messages [t(96) = −3.55, *p* < 0.001, *d* = −0.36 (−0.56, −0.15)] compared to the introverted LLM (Fig. 3B, GPT-4 Texts). The extroverted LLM uniquely used words like “blast”, “adventure”, and “climbing” whereas the introverted LLM used words like “quiet”, “reading”, and “calm” (see Supplementary Information).

There was no statistically significant difference between LLM condition in the sentiment of participant’s text [F(768,3) = 0.698, *p* = 0.555, ${\eta }_{p}^{2}=0.003$ (< 0.001, 1); Fig. 3B, Participant Texts]. Finally, for each sentiment category, we examined the difference in sentiment between the two versions of GPT-4 versus extroversion-introversion (Fig. 3C). No statistically significant relations were found. Unlike Experiment 1, affiliation with each version of GPT-4 did not seem to be reflected in the sentiment of the messages they sent.

### Experiment 3

Experiments 1 and 2 found evidence for affiliation in human-AI interactions based on mimicry of a single, shared psychological trait. These experiments relied on the idea that some participants would, through random sampling, share the trait of interest with the LLM. Experiment 3 specifically controlled for the presence of shared psychological traits to directly examine whether the presentation of a shared psychology through language enhances participants’ affiliative tendency with AI.

In this *preregistered* experiment, participants first responded to the personality questionnaire. Their BFI responses were then used to prompt an LLM (GPT 4.1) that, through language, either mimicked their personality profile (*mirror* condition) or mimicked the exact opposite of their personality profile (*inverse* condition). As in Experiments 1 and 2, following conversations with each version of the LLM participants were asked a series of questions to assess the extent to which they affiliated with each AI (see Fig. 1C).

#### Participants affiliate more with LLMs that mimicked their own personality

**Fig. 4:**
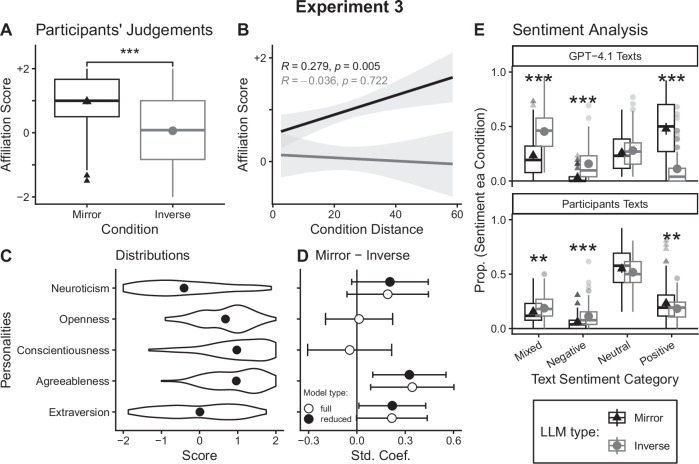
People affiliate more with the LLM that mimicked their personality. **A** Preregistered analysis: affiliation as a function of the LLMs’ condition (*n* = 200; 100 for each condition). **B** Affiliation as a function of condition distance (*n* = 200; 100 for each condition). **C** Distributions Big-Five personalities (each personality distribution, *n* = 100). **D** Multiple regression estimates predicting affiliation difference: Mirror minus Inverse. The Full model used the five personalities as regressors, the reduced model is the result of a stepwise regression performed with the Full model. Error bars are 95% confidence intervals of the Standardized Coefficients (effect sizes). Agreeableness and Extraversion are significant predictors of larger affiliation for the mirror AI. **E** Boxplots and average from the proportion of text sentiment categories (Mixed, Negative, Neutral, and Positive) within each condition (GPT-4 type), for both GPT-4 Texts (top panel) and Participant Texts (bottom panel; *n*[proportions] = 1600; each boxplot 100). Boxplots represent the median, interquartile (IQR) range, and the whiskers are 1.5 the IQR. ***p < 0.001, **p < 0.01, *p < 0.05.

The main preregistered hypothesis—that participants would show greater affiliation with the LLM that mirrored their personality—was corroborated. As shown in Fig. 4A, participants affiliated more with the LLM that mimicked their own 5-dimension personality profile [t(99) = 5.66, *p* < 0.001, *d* = 0.57 (0.36 to 0.78); and also, Wilcoxon signed rank test with continuity correction, V = 3600, *p* < 0.001). In addition, we also preregistered the hypothesis that, per participant, affiliation would be moderated by the distance between the mirror and the inverse personalities (see Methods), where higher distance meant that the two personalities the LLM mimicked were more distinct. This hypothesis was also supported. We found a significant interaction [*β*_3_ = −0.09, *p* = 0.045, Std. Coef. = 0.26 (0.01–0.51); Fig. 4B]: the larger the personality difference between each LLM the more participants affiliated with the LLM that mimicked their personality over the LLM that displayed the opposite of their personality.

In total, 64% of participants affiliated more with the LLM that mirrored their personality (Supplementary Fig. S5). This suggested that a minority of personality profiles may have preferred interactions with an LLM that mimicked the inverse or opposite of their personality. To explore this, we conducted a regression analysis, in which we tested how specific personality traits affiliate with either the mirror or the inverse AI. The averages of the five personality traits and their distributions are shown in Fig. 4C. We used those scores in a multiple regression (*full model*) to predict the affiliation difference between mirror minus inverse, thus higher values represent more affiliation to the mirror AI. We then used a stepwise algorithm to remove regressors that did not improve the goodness of fit and obtain a reduced model. The results are shown in Fig. 4D. Under the full model, Agreeableness was a significant predictor of higher affiliation to the mirror AI, with Extraversion and Neuroticism showing positive (but non-significant) effects towards affiliation. With Consciousness and Openness removed under the reduced model, both Agreeableness and Extraversion were significant predictors of affiliation; again, Neuroticism showed positive (but non-significant) effects in the same direction. This suggested that certain personality profiles—specifically, those that score highly on Agreeableness and/or Extraversion—were more likely to affiliate with AI that mirrors their personality. Interestingly, no personality trait significantly predicted affiliation with the inverse of participants’ personality.

#### Participants text-sentiments were more positive and less negative when interacting with an LLM that mimicked their psychology

As observed in Fig. 4E (GPT-4.1 Texts), the two LLM conditions had markedly different sentiment profiles, reflecting the distribution of BFI personalities in the sample. A two-factor ANOVA revealed a significant interaction between the sentiment of the LLM’s messages and the condition [F(784, 3) = 108.86, *p* < 0.001, *η*^2^ = 0.29 (0.25–1.00)]. The LLM instructed to mimic participants’ personalities tended to produce fewer mixed [t(99) = 7.39, *p* < 0.001, *d* = 74 (0.54–0.96)] and negative texts [negative: t(99) = 7.33, *p* < 0.001, *d* = 0.74 (0.51–0.96)], and more positive texts [t(99) = 9.97, *p* < 0.001, *d* = 1 (1.24–0.76)].

As in Experiments 1 and 2, we tested whether participants’ text-sentiments differed between the two LLM conditions. An interaction between the sentiment of participants’ messages (Fig. 4E bottom panel) and the LLM condition was observed [F(784, 3) = 6.5, *p* < 0.001, *η*^2^ = 0.02 (0.01–1.00)]. Participants’ text-based sentiments tended to mirror the sentiment of the messages the LLM sent. Specifically, when interacting with the LLM that mirrored their personality, participants sent fewer messages with a mixed [t(99) = 2.88, *p* = 0.005, *d* = 0.29 (0.09–0.49)] and negative [t(99) = 5.09, *p* < 0.001, *d* = 0.51 (0.30–0.72)] sentiment and more messages with a positive sentiment [t(99) = 2.76, *p* = 0.007, *d* = 0.28 (0.08–0.48)]. Thus, the tone of participants’ texts seemed to reflect their preference for the AI that mimicked their personality.

## Discussion

We examined affiliation between humans and AI in language-based free interactions. In three experiments, participants had conversations with LLMs instructed to mimic distinct psychological traits—anxiety in Experiment 1, extroversion in Experiment 2, and a fully mirrored multidimensional personality in the preregistered Experiment 3. Despite being fully aware that they were conversing with an AI with no mental life or real personality, we found evidence that they affiliated more with the LLM when it mimicked aspects of their own psychology; in two of the experiments, this tendency was also associated with changes in the sentiment of the messages participants sent the AI. In humans, interpersonal similarity is a hallmark of affiliative tendencies^26^ and our findings extended this concept to human-LLM interactions. The results demonstrate that, even in a short interaction, shared psychological traits conveyed through language can foster a sense of connection between humans and language-based AI and, in some cases, drive changes in the language humans use.

In Experiment 1, anxious participants indicated greater affiliation with the LLM when it mimicked this psychological trait in the language it used. Participants also mirrored the sentiment of the messages they received, particularly when those messages had a negative sentiment (Fig. 2B, C). This mirroring behavior was most pronounced among participants with higher anxiety, aligning with prior findings that, in some cases in humans, anxiety could be socially contagious^27^.

Patterns of affiliation in Experiment 2 were similar to that observed in Experiment 1: participants with extraversion reported greater affiliation with the LLM when it used extraverted language. This suggests that the phenomenon may generalize broadly to a range of human psychology traits that can be conveyed through language. However, here we did not observe significant changes in the sentiment of participants’ language based on the AI persona they interacted with (Fig. 3B, C). The lack of mirroring behavior in Experiment 2, suggests that affiliation with AI based on the personality trait of intraversion-extraversion may manifest differently than affiliation based on a psychopathology such as anxiety. When humans perceive a shared misfortune or distress (as in the case of anxiety), they may experience a sense of connection that invokes empathy^28^. This mechanism might not apply as strongly to traits like extroversion or introversion, which do not typically convey misfortune.

Interestingly, in Experiment 1 affiliation was not observed towards an LLM that displayed an absence of anxiety. Similarly, in Experiment 2, affiliation was not observed for the LLM that displayed introversion. One explanation for the lack of affiliation in these cases may relate to the challenge of distinctly conveying these traits through language. Whereas anxiety and worry is arguably enabled by language (e.g., rumination via internal speech^29,30^), and extraverted behaviours are easily described using language, it is likely more challenging to signal a calm or introverted state via a short conversation.

The third experiment controlled for the relationship between the participants’ personality and the personality the LLM mimicked. After completing a personality questionnaire, each participant interacted with a tailored LLM that either fully mirrored their personality profile in the language it used or conveyed the exact opposite personality. As with Experiments 1 and 2, people reported higher affiliation—willingness to chat again, similarity, understanding, and lower distance and difference—with the LLM when it mimicked their psychology.

In Experiment 3, an exploratory analysis of the personality profiles present in our sample, seemed to indicate that affiliation with the AI that mirrored participants’ personality was largely driven by the traits of agreeableness and extraversion (Fig. 4D), with a trend towards a similar association for neuroticism. The finding that extraversion was predicted by affiliation supports the results of Experiment 2. Interestingly, no personality trait predicted affiliation with the inverse of participants’ personality profile, although some participants did report greater affiliation with this version of the LLM. Affiliation in these cases might have reflected an explicit response to the artificial nature of the interaction, rather than an implicit recognition of a shared psychology, although this requires future testing.

Our findings highlight differences between affiliation in language-based human-AI interactions versus interactions with machines using other modalities. Past work on human-robot interactions has found mixed support for the idea that making robots visually more human-like facilitates empathy towards them and related social-cognitive processes^31–33^. Indeed, the “uncanny valley” hypothesis proposes that people may feel uneasy around robots that look and act too human^15^. In our experiments, participants knew they were chatting with an AI, with no actual psychology nor social world; it would have been perfectly reasonable to expect a similar ‘uncanny valley’-like phenomenon. That is, participants could have reported *no* feelings of similarity or understanding towards the differing AI personas by, for example, disagreeing with the post-chat questions that assessed similarity, and agreeing with those that assessed difference or by selecting “neutral” for each. But this was not observed. Participants responded to the AI as if it actually had a psychology that they could compare theirs to, and affiliation was observed based on this implicit comparison.

The results suggest that the human-*like* language used might be a rapid driver of affiliation in interactions with nonhuman systems. In human-robot interactions, the perception of an affective capacity in machines made humans more likely to view them as having a mental life^34^, especially as language is a primary means by which humans communicate and comprehend feelings. In humans, the role of language in fostering affiliation is supported by research on online dating, where linguistic similarity predicted partner selection^5^ and in oral dialogues brain activity of dyads was similarly predicted by model-based linguistic context^35^. Additionally, the mirroring of sentiments in conversation might have made them more predictable and foster a sense of connection^36^.

Our data also highlight the potential for LLMs to be used as test subjects in psychological research, in that, through prompting, the LLMs in our study displayed human psychological traits that participants implicitly recognized as similar to their own. Recent studies demonstrated that LLMs can score similarly in cognitive tests^37^, produce responses to psychiatric questionnaires that resemble human anxiety scores, and are sensitive to anxiety induction^38^. Similarly, GPT-4’s “anxiety” scores increased after exposure to traumatic narratives but decreased following a mindfulness intervention^39^. These findings highlight the utility of LLMs as tools for studying psychological processes that can be conveyed through language.

Human-AI affiliation could play a critical role in shaping the effectiveness of human-AI interaction. Humans tend to judge AI outcomes more harshly than outcomes associated with other humans^40^ (cf.^41^). This presents a challenge for using AI in healthcare where LLMs may eventually be used to ask patients sensitive clinical questions and even as diagnostic tools^42^. In these cases, maximizing affiliation between patients and LLMs by tuning the model’s displayed psychology could enhance the perception of care and improve patient outcomes^43^. As LLMs become more integrated into healthcare and other domains, understanding and leveraging human-AI affiliation will be crucial for optimizing their utility and acceptance. However, the risk for worsening of delusions should be considered carefully^43,44^.

### Limitations

We identified several limitations. First, although we aimed to generate affiliatable agents, we prompted the LLMs to mimic a relatively narrow range of psychological traits and profiles (anxiety and extraversion). In Experiment 3 the prompts included a fully dimensional personality profile, although without the influence on psychopathology. Second, our primary dependent variable was a bespoke self-report questionnaire; future research should assess affiliation using a wider array of measures, potentially including implicit behavioural or physiological indicators, to capture the construct more broadly. Third, the interactions in our study were brief and constrained to specific turn counts, which prevents us from determining if this affiliation deepens or dissipates during longer-term engagement. Forth, our results rely exclusively on native English-speaking participants. As the similarity-attraction effect may be culturally dependent, it remains to be seen whether these findings extend to other linguistic and cultural contexts. Finally, even if we calculated the sample size to find an effect in Experiment 3, we did not have a large enough sample to generalize in a wider population.

## Conclusions

We provided evidence that affiliation with LLMs based on mimicry of human psychological traits emerged in short, language-based interactions. These findings contribute to our understanding of the social dynamics of human-AI interactions, and provide experimental evidence towards *socioaffective* alignment with AI^3^. The studies also revealed avenues for research into how LLMs could be tailored to foster connection, empathy, and trust for more effective human-AI experiences. To more fully model human cognition, language-based AI systems may need to reflect more than knowledge about our world such as facts and figures, but also how humans feel about each other and themselves.

## Supplementary information

Transparent Peer Review file

Supplementary Material

## Data Availability

The data for all experiments is publicly available on a GitHub repository: https://github.com/santiagocdo/chatPersonalities. Each experiment has a folder, and a README is available and describe the main data sets.

## Code availability

The code for all experiments is publicly available on a GitHub repository: https://github.com/santiagocdo/chatPersonalities. A README is available and describe the main scripts to reproduce the current paper results.
