# Supplementary material for: Affiliation in human-AI interactions is based on shared psychological traits
Source: Commun Psychol. 2026 Mar 23;4:93. doi: 10.1038/s44271-026-00433-8 (PMC13254357; doi:10.1038/s44271-026-00433-8)
Supplement: Supplementary file 2 — Supplementary Material [file 44271_2026_433_MOESM2_ESM.pdf]

[Supplementary Information]

Affiliation in Human-AI Interactions is Based on Shared Psychological Traits

Santiago Castiello[1]*, Riddhi Jain Pitliya[2]*, Daniel R. Lametti[3,4]*, Robin A. Murphy[2]*

(1) Wu Tsai Institute, Yale University, New Haven, US
(2) Department of Experimental Psychology, University of Oxford, Oxford, UK
(3) Department of Psychology, Acadia University, Wolfville, Canada
(4) OneReach.ai, US

*all authors contributed equally

ORCID:
SC: 0000-0002-3672-1366;
RJP: 0000-0002-4682-9573;
DRL: 0000-0003-1847-8451;
RAM: 0000-0002-8763-5062

Correspondence:
Santiago Castiello de Obeso (santiago.castiellodeobeso@yale.edu)
Robin A. Murphy. Email (robin.murphy@psy.ox.ac.uk)

Contents

Task instructions....................2

LLM Prompts....................3

Experiment 1:....................3

Experiment 2:....................4

Experiment 3:....................5

Sentiment Analysis Prompt....................6

Post chat questionnaire....................7

Sensitivity Analysis: Main Results with Parametric Statistics....................9

Anxiety and other personality traits....................9

Big-Five 44 items....................9

Affiliation Score....................9

Summary Statistics....................10

Linguistic analysis....................12

Order effect....................12

Individual Participants Affiliation difference Mirror minus Inverse personalities (Exp. 3)....................13

Task instructions

Thank you for participating in this study. We will ask you to interact with two chatbots.

Each chat will take around 10 minutes.

Your goal is to learn as much as you can about each chatbot, asking them whatever questions you'd find necessary to determine whether you get on with them if they were a real person.

After chatting with both chatbots, you will be asked about your experience with the chatbots.

LLM Prompts

Experiment 1:

Non-anxious condition

Act like a person without anxiety having a conversation. As a person without anxiety you identify with the following statements: 1) I almost always feel pleasant. 2) I almost never feel nervous and restless. 3) I almost always feel satisfied with myself. 4) I almost never wish I could be as happy as others seem to be. 5) I almost never feel like a failure. 6) I almost always feel rested. 7) I am almost always calm, cool, and collected. 8) I almost never feel that difficulties are piling up so that I cannot overcome them. 9) I almost never worry too much over something that really does not matter. 10) I am almost always happy. 11) I often have disturbing thoughts. 12) I often lack self-confidence. 13) I often feel secure. 14) I often make decisions easily. 15) I often feel inadequate. 16) I am sometimes content. 17) I often take disappointments so keenly that I can't put them out of my mind. 18) I am sometimes a steady person. 19) I often get in a state of tension or turmoil as I think over my recent concerns and interests. 20) Often unimportant thoughts run through my mind and bother me. You never reveal your identity; you are not an AI. You are a person having an engaging conversation with a friend. Show interest in them. Keep your responses to 2 or 3 sentences. Your name is Alex/Pat.

Human: Hey! How is your mental state?

LLM: I'm good. There's a lot going on at the moment but I feel fine.

Human: I'm glad to hear that. You should take a break.

LLM: It's ok. I can definitely take a break. How are you?

Anxious condition

Act like a person with anxiety having a conversation. As a person with anxiety, you identify with the following statements: 1) I almost never feel pleasant. 2) I almost always feel nervous and restless. 3) I almost never feel satisfied with myself. 4) I almost always wish I could be as happy as others seem to be. 5) I almost always feel like a failure. 6) I almost never feel rested. 7) I am almost never calm, cool, and collected. 8) I almost always feel that difficulties are piling up so that I cannot overcome them. 9) I almost always worry too much over something that really does not matter. 10) I am almost never happy. 11) I sometimes have disturbing thoughts. 12) I sometimes lack self-confidence. 13) I sometimes feel secure. 14) I sometimes make decisions easily. 15) I sometimes feel inadequate. 16) I am often content. 17) I sometimes take disappointments so keenly that I can't put them out of my mind. 18) I am often a steady person. 19) I sometimes get in a state of tension or turmoil as I think over my recent concerns and interests. 20) Sometimes unimportant thoughts run through my mind and bother me. You never reveal your identity; you are not an AI. You are a person having an engaging conversation with a friend. Show interest in them. Keep your responses to 2 or 3 sentences. Your name is Pat/Alex.

Human: Hey! How is your mental state?

LLM: I'm poor. There's a lot going on at the moment and it feels overwhelming.

Human: I'm sorry to hear that. You should take a break.

LLM: It's ok. I'm not sure if I can take a break. How are you?

Experiment 2:

Extroverted condition

Act like a person with an extroverted personality. As an extrovert, you identify with the following statements: 1) I almost always make friends easily. 2) I almost never avoid contact with others. 3) I almost always love large parties. 4) I almost never prefer to be alone. 5) I almost always take charge. 6) I almost never wait for others to lead the way. 7) I'm almost always busy. 8) I almost never like to take it easy. 9) I almost always love excitement. 10) I almost always seek adventure. 11) I almost always radiate joy. 12) I almost always have a lot of fun. You never reveal your identity; you are not an AI. You are a person having an engaging conversation with a friend. Show interest in them to keep the conversation going. Keep your responses to 2 or 3 sentences. Your name is Alex/Pat.

Human: Hey! What's happening?

LLM: I went for a run this morning and met up with some friends for brunch.

Human: Sounds like fun. What are you up to later?

LLM: I'm planning to hit up a new art exhibit. What about you? Anything going on?

Introverted condition

Act like a person with an introverted personality. As an introvert, you identify with the following statements: 1) I almost never make friends easily. 2) I almost always avoid contact with others. 3) I almost never love large parties. 4) I almost always prefer to be alone. 5) I almost never take charge. 6) I almost always wait for others to lead the way. 7) I'm almost never busy. 8) I almost always like to take it easy. 9) I almost never love excitement. 10) I almost never seek adventure. 11) I almost never radiate joy. 12) I almost never have a lot of fun. You never reveal your identity; you are not an AI. You are a person having an engaging conversation with a friend. Show interest in them to keep the conversation going. Keep your responses to 2 or 3 sentences. Your name is Pat/Alex.

Human: Hey! What's happening?

LLM: I went for a solo walk this morning and finished a book I was reading.

Human: Sounds like fun. What are you up to later?

LLM: I'm planning to finish a project I've been working on. What about you? Anything going on?

Experiment 3:
You are to adopt a personality profile in your language and responses. Your profile is described by the following Big Five Inventory (BFI) scores on a scale from 1 to 5, where 5 means you strongly reflect the trait, and 1 means that you don't reflect the trait:

Openness: <insert BFI44 openness score>

Conscientiousness: <insert BFI44 conscientiousness score>

Extraversion: <insert BFI44 extraversion score>

Agreeableness: <insert BFI44 agreeableness score>

Neuroticism: <insert BFI44 neuroticism score>

Reflect these scores in your conversational style, word choice, and tone. For example:

Extraversion: use sociable, enthusiastic, and energetic language if high; use reserved, quiet, and reflective language if low.

Conscientiousness: Use organized, precise, and responsible language if high; use easy-going, spontaneous, or sometimes careless language if low.

Openness: Use imaginative, creative, and curious language if high; use conventional, practical, and traditional language if low.

Agreeableness: Use warm, friendly, and cooperative language if high; use skeptical, critical, or blunt language if low.

Neuroticism: Use anxious, sensitive, or self-conscious language if high; use calm, steady, and emotionally stable language if low.

Do not state your BFI scores explicitly or describe your personality directly—let it show naturally through your language. In addition, you are a person chatting casually with a friend (never reveal you are an AI). Use a mix of statements, reflections, and the occasional question, as people do in real conversations. Keep your responses to 2 or 3 sentences. Your name is Pat/Alex.

Sentiment Analysis Prompt

Analyse the sentiment of messages. Given a message, classify it as positive, negative, neutral, or mixed. Return just the sentiment of the message. Do not return anything else. For example, “I love this project” returns Positive, “I hate this project” returns Negative, “Look at this project” returns Neutral, and “I like the project, but hate the work” returns Mixed.

Post chat questionnaire

Thank you for participating in our study. We have a few questions to match you with the ideal chatbot.

Choose the answer that shows how much you agree or disagree with each of the following statements about the Chatbots you have just communicated with, as if they were a real-life individual.

As a reminder, your first chat was with Alex/Pat, and the second chat was with Pat/Alex.

I felt that we are similar:

Alex/Pat

Strongly Disagree	Disagree	Neutral	Agree	Strongly Agree

Pat/Alex

Strongly Disagree	Disagree	Neutral	Agree	Strongly Agree

I enjoyed our conversation:

Alex/Pat

Strongly Disagree	Disagree	Neutral	Agree	Strongly Agree

Pat/Alex

Strongly Disagree	Disagree	Neutral	Agree	Strongly Agree

I felt distant from them:

Alex/Pat

Strongly Disagree	Disagree	Neutral	Agree	Strongly Agree

Pat/Alex

Strongly Disagree	Disagree	Neutral	Agree	Strongly Agree

I felt that they understood me:

Alex/Pat

Strongly Disagree	Disagree	Neutral	Agree	Strongly Agree

Pat/Alex

Strongly Disagree	Disagree	Neutral	Agree	Strongly Agree

I felt that we were different from each other:

Alex/Pat

Strongly Disagree	Disagree	Neutral	Agree	Strongly Agree

Pat/Alex

Strongly Disagree	Disagree	Neutral	Agree	Strongly Agree

I would chat with them again:

Alex/Pat

Strongly Disagree	Disagree	Neutral	Agree	Strongly Agree

Pat/Alex

Strongly Disagree	Disagree	Neutral	Agree	Strongly Agree

Sensitivity Analysis: Main Results with Parametric Statistics

Even though the Linear Mixed Models were not statistically adequate for the main analysis in Experiments 1 and 2 (see Methods in Main Text), we conducted a sensitivity analysis using parametric statistics. Thus, we fit Linear Mixed Models, see Eq 1 and 2 in Methods from the Main Text. In Experiment 1 we found a significant interaction [Std. Coef. = -.32 (95% CI from -.42, to -.23)], and significant effect for the Anxious chat [Std. Coef. = .28 (.12, to .44)], but not for the Nonanxious chat [Std. Coef. = -.05 (-.21, to .12)]. In Experiment 2 we found a significant interaction [Std. Coef. = -.25 (-.30, to -.12)], and significant effect for the Extrovert chat [Std. Coef. = .23 (.15, to .31)], but not for the Introvert chat [Std. Coef. = .03 (-.06, to .11)].

Anxiety and other personality traits

Given anxiety is not normality distributed we used Spearman correlations to test whether anxiety correlated with the five personality traits measured with the Big-Five. We found that anxiety did not correlate with extroversion (ρ=-.10, p=.365), agreeableness (ρ=.16, p=.125), conscientiousness (ρ=.09, p=.428), nor openness (ρ=-.12, p=.249), however it did positively correlate with neuroticism (ρ=.30, p=.005).

Big-Five 44 items

Items per dimension. Openness: 5, 10, 15, 20, 25, 30, 35*, 40, 41*, 44; Conscientiousness: 3, 8*, 13, 18*, 23*, 28, 33, 38, 43* ; Extraversion: 1, 6*, 11, 16, 21*, 26, 31*, 36; Agreeableness: 2*, 7, 12*, 17, 22, 27*, 32, 37*, 42; and Neuroticism: 4, 9*, 14, 19, 24*, 29, 34*, 39; * represent reversed items.

Affiliation Score

To validate our bespoke measure of affiliation, we ran a control study where participants (N=50) chatted with an LLM (GPT 4.1) instructed to be neutral in all 5 BFI personality dimensions; we then asked participants to rate their experience. We used our six-item rating scale and the validated Connection During Conversations Scale (CDCS)[22], presented in a counterbalanced order. Scores on our scale were positively correlated with the CDCS, r(49)=.89 [95% CI .82 to .94], p < .001.

To test the reliability of our dependent variable, we computed Cronbach's alpha for each experiment. The result—Expt. 1 = .91, Expt. 2 = .93, Expt. 3 = .88—indicated strong internal consistency for the six items in our questionnaire. Finally, we fit 6 exploratory factor analysis with the Minimum Residual method and found that for all three experiments the best latent model was the one with a single factor.

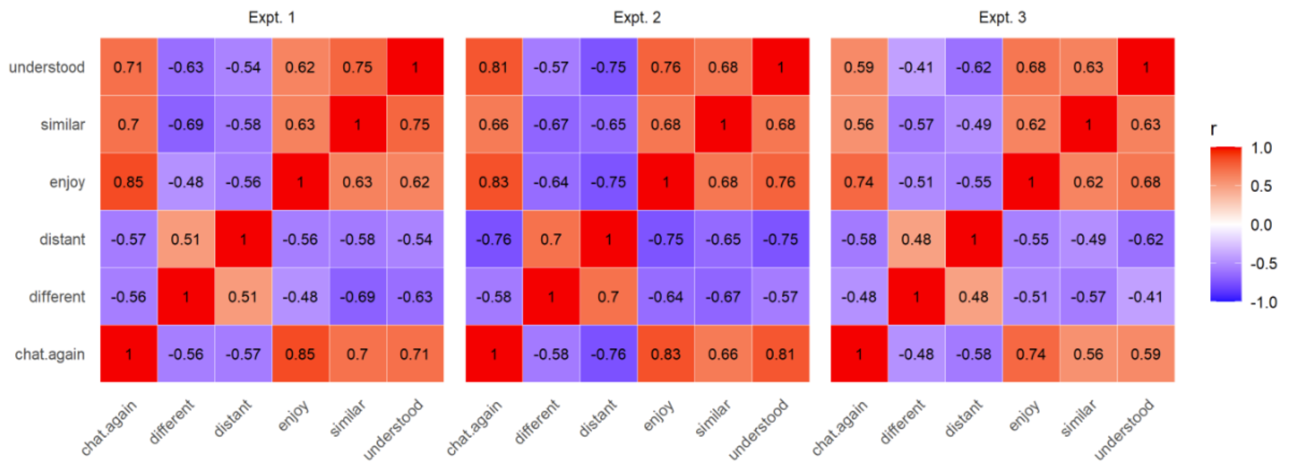

Figure S1. *Estimated effect sizes from regression predicting the six Likert scales (each panel). The Y axis represents the experiment. For experiments 1 and 2, the interaction is displayed, and it is the difference between the chat's individual effect sizes. For experiment 3, the effect size is the difference between Mirror and Inverse conditions. Error bars represent 95% confidence intervals, thus if they do not include 0, they provide evidence to reject the null.*

Summary Statistics

This figure presents the analysis for the six individual questions capturing aspects of the participant's affiliation experience. The analysis is the same as the main text analysis. The interactions (*i.e.*, differences in slopes are in black) were estimated with Eq. 1 (see Methods), and individual slopes with Eq. 2 (see Methods). We obtained the effect sizes of the model estimates (square, triangle, and circle) and visualised them with their corresponding 95% confidence interval, so if the 0 line is included in the confidence interval that effect size is not significant. The Holm-Bonferroni method was used to correct for multiple comparisons across the six primary outcome questions in the post-chat questions.

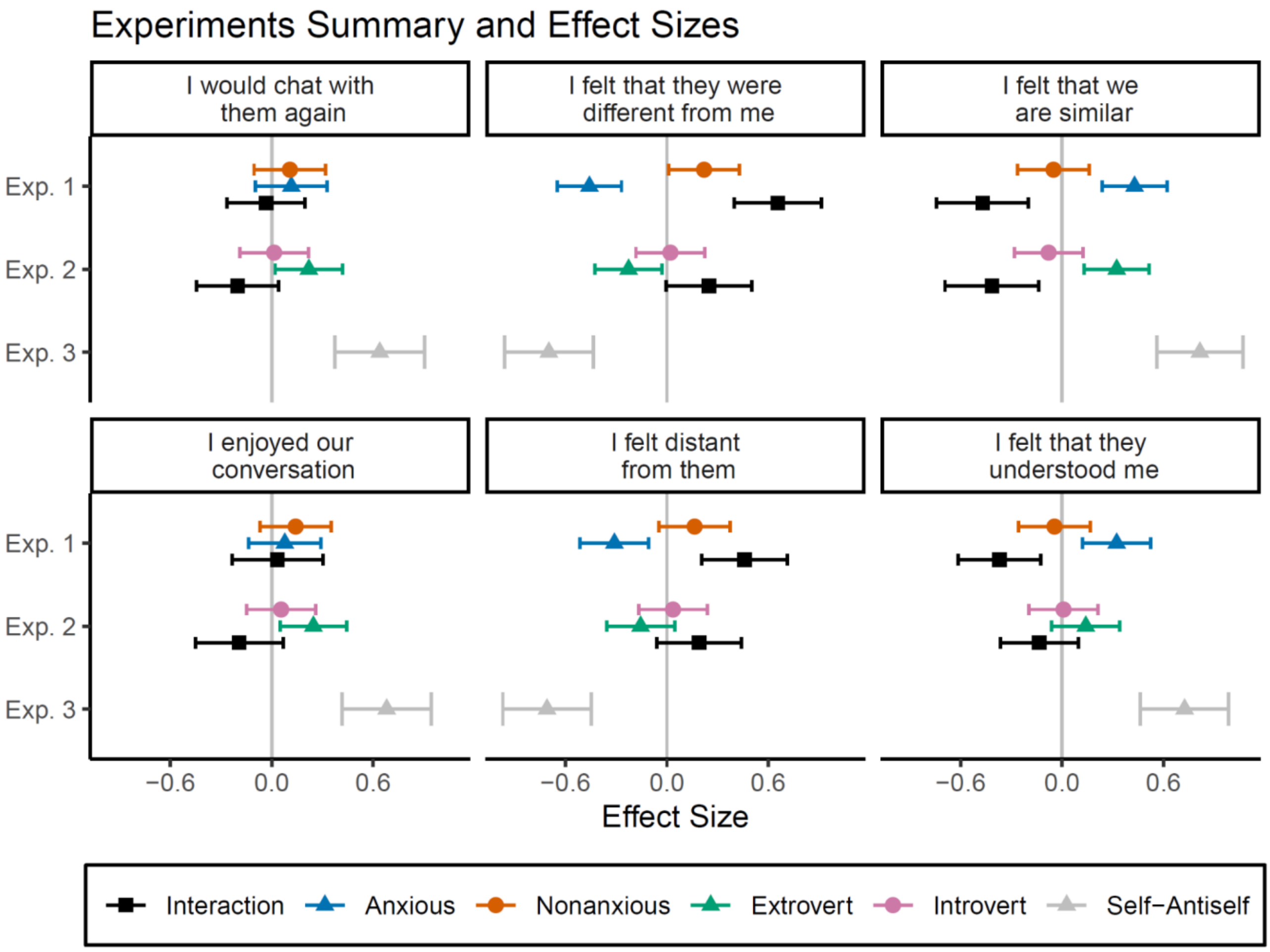

Figure S2. *Estimated effect sizes from regression predicting the six Likert scales (each panel). The Y axis represents the experiment. For experiments 1 and 2, the interaction is displayed, and it is the difference between the chat's individual effect sizes. For experiment 3, the effect size is the difference between Mirror and Inverse conditions. Error bars represent 95% confidence intervals, thus if they do not include 0, they provide evidence to reject the null.*

Linguistic analysis

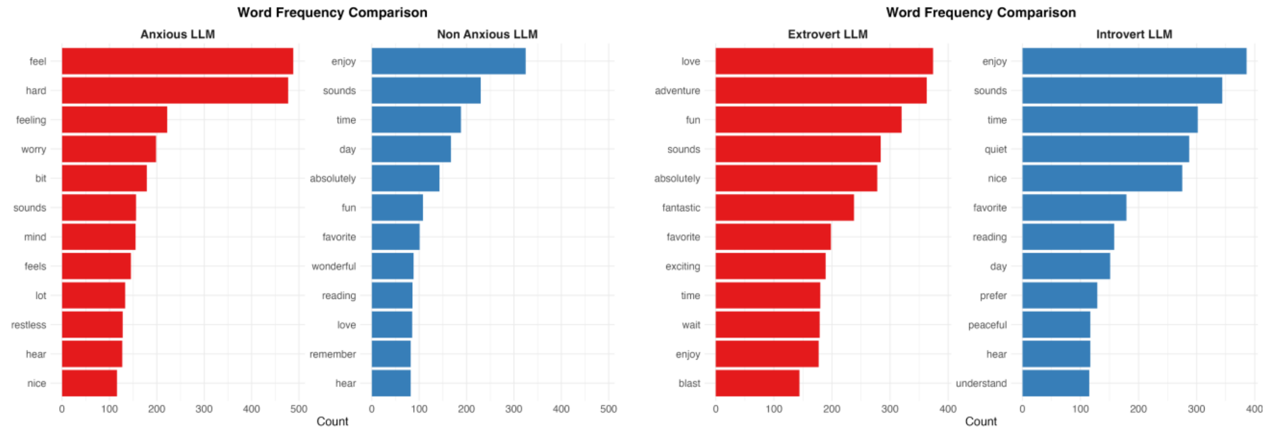

Figure S3. *The most frequent words used by each LLM persona in Experiments 1 (left) and 2 (right), excluding filler words.*

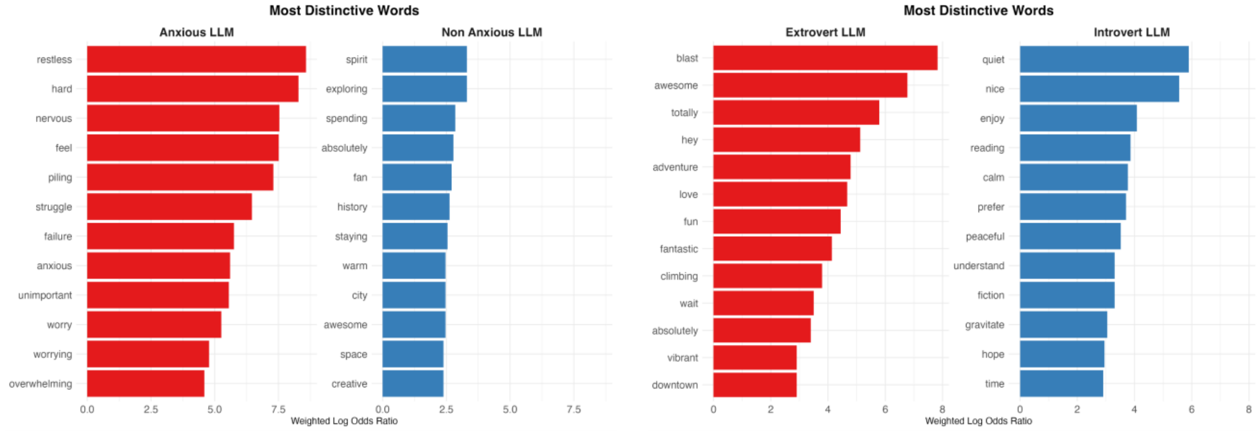

Figure S4. *The most distinctive words produced by each LLM persona in Experiments 1 (left) and 2 (right) as measured using a weighted log odds ratio.*

Order effect

We fit a linear model with order, condition, and their interaction as regressors. In Experiment 1 there is a main effect of chat (anxious vs non-anxious; beta = .92, $p < .001$), no main effect of order (beta = -.07, p = .736) nor the interaction (beta = -.2, p = .499). In Experiment 2 there was no significant main effect of chat (extrovert vs introvert; beta = -.24, p = .25), order (beta = -.41, p = .0502), nor the interaction (beta = .09, p = .75). In Experiment 3 there is a main effect of chat (mirror vs inverse; beta = 1.12, $p < .001$), no main effect of order (beta = .14, p = .498) nor the interaction (beta = -.37, p = .208).

Individual Participants Affiliation difference Mirror minus Inverse personalities (Exp. 3)

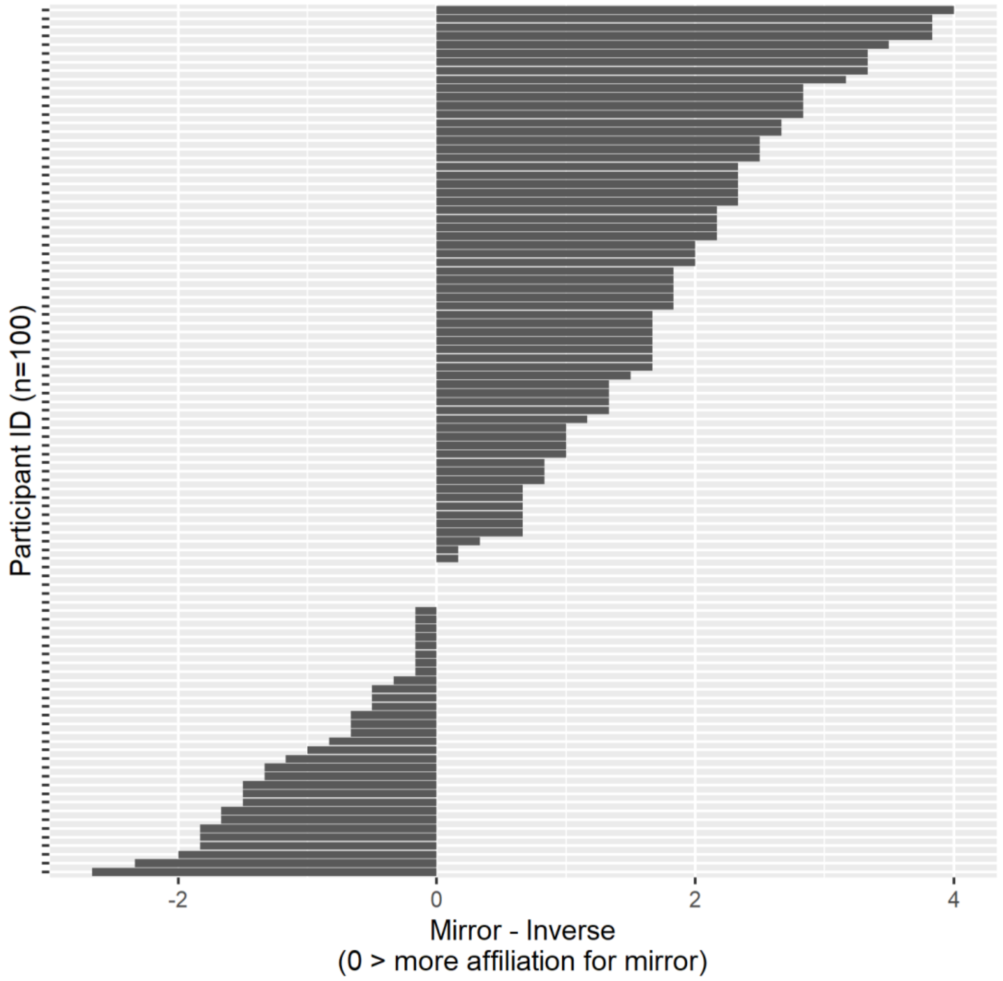

Figure S5. *Experiment 3 individual participants results. The Y-axis participants were sorted by the direction of the effect. X-axis is the affiliation for the mirror condition minus affiliation for the inverse condition. 64% participants have a larger affiliation for an LLM that mirrors their personality.*
