# Supplementary material for: Affiliation in human-AI interactions is based on shared psychological traits
Source: Commun Psychol. 2026 Mar 23;4:93. doi: 10.1038/s44271-026-00433-8 (PMC13254357; doi:10.1038/s44271-026-00433-8)
Supplement: Supplementary file 1 — Transparent Peer Review file [file 44271_2026_433_MOESM1_ESM.pdf]

nature portfolio

Peer Review File

Affiliation in Human-AI Interactions is Based on Shared Psychological Traits

Corresponding Author: Dr Santiago Castiello

This file contains all editorial decision letters in order by version, followed by all author rebuttals in order by version.

Version 0:

Decision Letter:

** Please ensure you delete the link to your author homepage in this e-mail if you wish to forward it to your coauthors **

Dear Dr Castiello,

Thank you for your patience during the peer-review process. Your manuscript titled "Affiliation in human-AI interactions based on shared psychological traits" has now been seen by 3 reviewers, whose comments are appended below. You will see that they find your work of some potential interest. However, they have raised quite substantial concerns that must be addressed. In light of these comments, we cannot accept the manuscript for publication, but would be interested in considering a revised version that fully addresses these serious concerns.

We hope you will find the Reviewers' comments useful as you decide how to proceed. Should additional work allow you to address these criticisms, we would be happy to look at a substantially revised manuscript. If you choose to take up this option, please highlight all changes in the manuscript text file, and provide a detailed point-by-point reply to the reviewers.

Please bear in mind that we will be reluctant to approach the reviewers again in the absence of substantial revisions.

In particular, two reviewers raise serious concerns about the construct validity of your main outcome measure. The bespoke six-item “affiliation” scale is unvalidated, leaving uncertainty about whether it captures the intended construct or aligns with established measures of liking or connection. Validation of this measure or inclusion of validated alternatives is essential to strengthen your conclusions.

Further, the manipulation of chatbot personality traits is not empirically verified. The absence of a manipulation check limits interpretability of both significant and null effects. You should include or report evidence that the AI expressed the intended traits, whether via human ratings, linguistic analysis, or other methods.

The manuscript also needs clearer reporting on preregistration, exclusion criteria, and sample determination, as well as full statistical reporting of effects and effect sizes. Reviewers also found the theoretical positioning relative to existing similarity–attraction research underdeveloped. Strengthening this section will clarify your contribution beyond prior work.

Please ensure you follow our statistical guidelines when reporting statistics (https://www.nature.com/commspsychol/submit/submission-guidelines#statistical-guidelines). Please note in particular our requirements for the reporting and interpretation of null-results. Non-significant findings derived from null-hypotheses significance tests should be reported in full, but may not be interpreted. Where you interpret null results, this interpretation must be based on Bayes Factors or equivalence tests.

I am attaching a checklist that details critical reporting requirements for the revised manuscript. Please attend to each item and ensure your manuscript is fully compliant. We are requesting that your manuscript aligns with these requirements as this facilitates the evaluation of your manuscript, reducing delays in re-review and potential future acceptance. If your revised manuscript is not aligned with these requests on major issues, such as those concerning statistics, it may be returned to you for further revisions without re-review. Additional information can be found in our style and formatting guide Communications Psychology formatting guide.

If the revision process takes significantly longer than five months, we will be happy to reconsider your paper at a later date, provided it still presents a significant contribution to the literature at that stage.

We would appreciate it if you could keep us informed about an estimated timescale for resubmission, to facilitate our planning.

We are committed to providing a fair and constructive peer-review process. Please do not hesitate to contact us if you wish to discuss the revision in more detail.

Please use the following link to submit your
- revised manuscript,
- point-by-point response to the referees' comments,
- cover letter (as a separate document),
- the Reporting Summary (see below), and
- the completed Editorial Request Table (attached):

Link Redacted

** This url links to your confidential home page and associated information about manuscripts you may have submitted or be reviewing for us. If you wish to forward this email to co-authors, please delete the link to your homepage first **

Thank you for the opportunity to review your work.

Best regards,

Anna-Lena Schubert

Anna-Lena Schubert, PhD
Editorial Board Member
Communications Psychology
orcid.org/0000-0001-7248-0662

REVIEWER EXPERTISE:
Reviewer #1 human/ai interaction
Reviewer #2 human/ai interaction
Reviewer #3 human/ai interaction

REVIEWER REPORTS:

Reviewer #1 (Remarks to the Author):

It's a carefully done, important, timely study. It should be published. I just thought some issues could be made a little clearer earlier on in the manuscript. Specific comments below.

Page 1, abstract. It might be helpful to define what it would mean for an LLM to exhibit the 'opposite' of someone's personality

Line 86 – examples of clinically relevant criteria here would be helpful rather than just directing the reader to a reference

Line 89. Why was their anxiety measured after the conversation rather than before? Is there a chance that the conversation had a priming effect of some sort? Was it trait or state anxiety that was measured?

Line 95. Again, what 'validated tests of personality' were used – more specifics/examples would be useful and appropriate.

Line 98. So the prediction was only about extroversion? Also, was this 'predicted' in advance as in a pre-registered hypothesis, or it's just informally what you expected might happen but you didn't pre-register that it would?

Line 103. What is the 'inverse' of a person's personality profile?

Line 124. Why did you settle on 100 to recruit?

Line 125. Were the exclusion criteria registered?

Line 133. You recruited 100 b/c you anticipated exclusions?

Line 151. Extra comma after "included"

Line 157. Maybe I missed it, but were participants explicitly told they'd be chatting with an AI? Or did they think they were chatting with a human? The introduction of Turing's test in the introduction might make it seem like this is a set up where they don't know but are supposed to guess whether it's a human or an LLM they're conversing with. In any case, this key fact should be made clear in the beginning and be much more prominent.

Line 172. Why as 8 turns chosen as the exclusion criterion, and was this registered?

Line 184. First time I'm seeing the information that participants were told they were interacting with chatbots - this is really key information needs to be highlighted earlier.

Line 222 – something going on with the equation/formatting

Lien 252. Is this assumption about Likert scales appropriate? Were the answers in fact normally distributed? Also, Likert scales are not continuous.

Reviewer #2 (Remarks to the Author):

The work describes 3 empirical studies (one pre-registered) that GPT is perceived as eliciting greater affiliation when it matches the participant's personality. This is timely given to power and potential of LLMs to manipulate. The study is generally well-executed, but several methodological concerns must be addressed to make the results interpretable and convincing. The novelty appears limited given prior work on this topic, and the work needs to better position its novel contribution in light of prior work on social robots and agents. Some discussion of ethics seems necessary. The writing and design is clear with minor caveats.

NOVELTY: The author's claim that evidence for the similarity-attraction theory in agents has "little empirical support." However, there is extensive empirical evidence going back to work by Cliff Nass in the '90s and extending to the present day. This includes at least one meta-analysis on the topic. The authors do not cite or seem aware of this literature. While the paper may address limitations in this prior work, the novel contribution must be clarified. Some examples
- Moon and Nass 1996: Finds results consistent with similarity-attraction theory related to dominance.
- Nass and Lee 2001: showed similarity-attraction effects for extroversion
- Esterwood et al 2021: Mena-analysis of 13 similarity-matching studies in HRI

Note also the study used a single context (open-ended dialog) and a single measure of success (an unvalidated bespoke scale), lowering the novelty and generality of the results.
-
METHODOLOGY: There are a number of conceptual and methodological issues with the work that would need to be addressed before the work is suitable for publication. At minimum, these would require re-analysis and clarifying how these analyses relate to the pregistered plan. It would likely require at least one new experiment.

Manipulation check: The authors prompt GPT to express a personality but the success of this prompting is never validated. Examples of validation would include recruiting a separate pool of human annotators or using pre-trained personality recognition models or linguistic analysis like LIWC. Though less convincing, this could include using another LLM to code dialogs. This is particularly important in explaining the failure to support the hypothesis when the trait is prompted to be low. For example, whereas we see the similarity-attraction effect when the model is prompted to be anxious or extroverted, we see no effect when the model is prompted to be non-anxious or introverted. The null findings in the low-trait conditions deserve more explicit discussion.
Problematic measure of attraction: the use of a "bespoke" measure is puzzling, given that there are many validated measures that could have been used. Given you innovate your own scale, you must report scale reliability (e.g, Cronbach's alpha). If the scale is not reliable, then you may wish to perform factor analysis, though your N is probably too low for this.

Reliance on within-subjects design: The use of a within-subjects design, and especially the choice to provide the scale only after interacting with both agents, will tend to amplify the effect and can lead to experimenter effects (e.g., when participants realize the goal of the study). This seems not ecologically valid as real users would not likely face such a stark choice in the real world. This should be discussed but a stronger paper would include a pre-registered Study 4 that uses a between-subjects design.

Failure to analyze order effects: The use of a within-subjects design creates order effects. These should be analyzed and reported as they might change the interpretation of the results.

Incomplete reporting of results: The analysis should report the significance of the main effects and interaction terms (i.e., the sig of the coefficients corresponding to these factors). Also report effect sizes. Are these interesting effects?

Other issues:
- The nature of the task was hard to discern from the main text. It would help to have the exact instructions. Given the description, and that participants make the first dialog move, it seems possible there is considerable variance in the conversations
- Objective analysis of the text might help unpack causality as to who is influencing who. Right now, some results are described as causal (e.g, participant adapts to agent) when they are simply correlational (e.g., discussion of fig 2b&c)
- The description of how experiment implemented hard to follow and seems to assume some familiarity with GSX. They way that long dialog contexts are handled is particularly unclear (Exp 1&2).
- I would suggest against calling out specific BFI items in the description of Study 3: this goes in supplemental. Just describe conceptually.
- Focus on the uncanny valley seems odd and distracts from your point. You never assessed if dialog seems human-like of uncanny. Your presumption is they are all human-like but you are simply manipulating style (note there is plenty of literature suggesting that LLMs have a default personality – e.g., agreeable) so it is not like you are explicitly trying to make them more/less human-like.

In sum, this work raises interesting questions about personality matching in LLM-mediated interaction, but its methodological and theoretical framing require substantial revision before it can make a reliable contribution to the literature

Jonathan Gratch, USC

Reviewer #3 (Remarks to the Author):

This is a review of the manuscript entitled "Affiliation in human-AI interactions based on shared psychological traits." The paper is timely, methodologically solid, and relatively well-written. A key strength of the paper is the fact that the authors take a well-established psychological phenomenon (similarity leads to affiliation and liking) and examine whether this phenomenon applies when people chat with an LLM-powered chatbot. The paper's findings thus have theoretically-grounded implications for understanding human-AI interactions and relationships, as well as practical applications for designing AI that is better able to facilitate rapport between humans and AI agents. Previous research does suggest that mimicry, when too obvious, can backfire, so the findings of the paper, while not surprising, are far from obvious either. Overall, this paper is a good example of how new phenomena, like LLM-powered chatbots, can be rigorously studied and connected to established psychological theories and findings.

All of that said, I do have one concern that I would classify as major. Specifically, the key outcome measure is an ad-hoc, or bespoke, measure of six items. While face-valid, this measure is not validated or used in previous research. Thus, it is not clear how the findings of this paper really relate to the more established findings around similarity, liking, and affiliation. This is not a minor issue because ultimately, without grounding and validation, it is not clear what we are measuring, whether or not we can call it affiliation, or how it relates to other constructs. For example, in the Discussion, the authors seem to be interpreting the finding as an indicator of a sense of connection that humans experience with the AI ("…through language could foster a sense of connection between humans and AI."). But there are established measures of liking and sense of connection that are not employed here, so we do not know if this is the correct interpretation of the findings.

I can see several ways that this issue can be resolved. First, the authors could run a "pilot study" where they validate this measure against a range of established measures of connection, liking, and affiliation. This will make the existing studies more convincing. Second, the authors could re-run one of the studies (e.g., Study 3) with more measures that are established and validated. At the very least, though, this issue needs to be addressed head-on in the Discussion. Relatedly, the Discussion is very sparse on acknowledging limitations of the study (e.g., MTurk sample, findings that did not confirm hypotheses, use of unvalidated measures, and so on).

The decision to exclude certain participants in Studies 1 and 2 without that decision or criteria being preregistered warrants more explanation. How were the exclusion criteria derived? What measures were taken to ensure that the criteria were not influenced by the results of the study (e.g., were they set before looking at the data)? I would prefer seeing the results without excluding participants, at least in SOM.

There are some typos throughout the manuscript.

The writing is generally clear. However, the second paragraph, where the Turing test is talked about, is not well integrated into the narrative, and it is not clear how the Turing test has anything to do with the subject matter of the paper (or at least why it deserves an entire paragraph). Some sentences are unclear and need revision. For example, I have a hard time understanding what the following sentence means: "But the degree to which people feel more similar to an AI that reflects characteristics of their own psychology remains unknown." Finally, the Methods section was quite difficult to understand because statistical tests are described without explaining what question each statistical test would answer. This becomes a bit clearer in the Results, but it needs to be clearer why certain tests are being performed and how they build on each other

to address the RQs.

Finally, in Studies 1 and 2, the hypotheses seem only partially supported. Specifically, people’s own traits are not related to how affiliated they feel with the non-anxious and introverted chatbots. Why is that? As far as I can tell, this finding is not predicted. Is there a reason the traits are only related to affiliation for the anxious and extroverted bots? More discussion is needed on this point, and it needs to be acknowledged as a limitation (since this pattern was not predicted and appears inconsistent with the hypothesis).
I hope my review is helpful. I sign my reviews: Kostadin Kushlev

EDITORIAL POLICIES

We ask that you ensure your manuscript complies with our editorial policies and reporting requirements.

To that end, we require revised manuscripts to be accompanied by a completed item: a reporting summary that collects information on study design and procedure.

- Nature Research Reporting Summary

Your revised manuscript can only be sent back to the referees if this checklist is completed and uploaded with the revision.

Notes: If you have submitted a Stage 1 Registered Report, Review, Primer, Comment, or Perspective you do not need to submit these forms. If you have already submitted these forms, you may disregard this request.

* TRANSPARENT PEER REVIEW: Communications Psychology uses a transparent peer review system. This means that we publish the editorial decision letters including Reviewers' comments to the authors and the author rebuttal letters online as a supplementary peer review file. However, on author request, confidential information and data can be removed from the published reviewer reports and rebuttal letters prior to publication. If your manuscript has been previously reviewed at another journal, those Reviewers' comments would not form part of the published peer review file.

** Visit Nature Research's author and referees' website at www.nature.com/authors for information about policies, services and author benefits**

Communications Psychology is committed to improving transparency in authorship. As part of our efforts in this direction, we are now requesting that all authors identified as ‘corresponding author’ create and link their Open Researcher and Contributor Identifier (ORCID) with their account on the Manuscript Tracking System prior to acceptance. ORCID helps the scientific community achieve unambiguous attribution of all scholarly contributions. You can create and link your ORCID from the home page of the Manuscript Tracking System by clicking on ‘Modify my Springer Nature account’ and following the instructions in the link below. Please also inform all co-authors that they can add their ORCIDs to their accounts and that they must do so prior to acceptance.
https://www.springernature.com/gp/researchers/orcid/orcid-for-nature-research

For more information please visit http://www.springernature.com/orcid

If you experience problems in linking your ORCID, please contact the Platform Support Helpdesk.

Version 1:

Decision Letter:

** Please ensure you delete the link to your author homepage in this e-mail if you wish to forward it to your coauthors **

Dear Dr Castiello,

Your manuscript titled "Affiliation in human-AI interactions based on shared psychological traits" has now been seen by our reviewers, whose comments appear below. In light of their advice I am delighted to say that we are happy, in principle, to

publish a suitably revised version in Communications Psychology.

We therefore invite you to revise your paper one last time to address the remaining concerns of our reviewers and a list of editorial requests. At the same time we ask that you edit your manuscript to comply with our format requirements and to maximise the accessibility and therefore the impact of your work.

EDITORIAL REQUESTS:

Please review our specific editorial comments and requests regarding your manuscript in the attached "Editorial Requests Table". Please outline your response to each request in the right hand column. Please upload the completed table with your manuscript files as a Related Manuscript file.

If you have any questions or concerns about any of our requests, please do not hesitate to contact me.

SUBMISSION INFORMATION:

In order to accept your paper, we require the files listed here https://www.nature.com/documents/commsj-file-checklist.pdf .

OPEN ACCESS:

Communications Psychology is a fully open access journal. Articles are made freely accessible on publication. For further information about article processing charges, open access funding, and advice and support from Nature Research, please visit https://www.nature.com/commspsychol/open-access

At acceptance, you will be provided with instructions for completing the open access licence agreement on behalf of all authors. This grants us the necessary permissions to publish your paper. Additionally, you will be asked to declare that all required third party permissions have been obtained, and to provide billing information in order to pay the article-processing charge (APC).

* TRANSPARENT PEER REVIEW: Communications Psychology uses a transparent peer review system. On author request, confidential information and data can be removed from the published reviewer reports and rebuttal letters prior to publication. If you are concerned about the release of confidential data, please let us know specifically what information you would like to have removed. Please note that we cannot incorporate redactions for any other reasons.

* CODE AVAILABILITY: All Communications Psychology manuscripts must include a section titled "Code Availability" at the end of the methods section. We require that the custom analysis code supporting your conclusions is made available in a publicly accessible repository at this stage; please choose a repository that generates a digital object identifier (DOI) for the code; the link to the repository and the DOI must be included in the Code Availability statement. Publication as Supplementary Information will not suffice.

* DATA AVAILABILITY:
All Communications Psychology manuscripts must include a section titled "Data Availability" at the end of the Methods section. More information on this policy, is available in the Editorial Requests Table and at http://www.nature.com/authors/policies/data/data-availability-statements-data-citations.pdf.

Please use the following link to submit the above items:
Link Redacted
** This url links to your confidential home page and associated information about manuscripts you may have submitted or be reviewing for us. If you wish to forward this email to co-authors, please delete the link to your homepage first **

We hope to hear from you within two weeks; please let us know if you need more time.

Best regards,

Jennifer Bellingtier

Jennifer Bellingtier, PhD
Senior Editor
Communications Psychology

Anna-Lena Schubert, PhD
Editorial Board Member

Communications Psychology
orcid.org/0000-0001-7248-0662

REVIEWER EXPERTISE:
Reviewer #1 human/ai interaction
Reviewer #2 human/ai interaction
Reviewer #3 human/ai interaction

REVIEWERS' COMMENTS:

Reviewer #1 (Remarks to the Author):

I think the revision is adequate. Thank you.

Reviewer #2 (Remarks to the Author):

I appreciate the efforts to address my concerns and related concerns by R3. I feel these have been adequately addressed and I'm happy to recommend the paper for acceptance.

Reviewer #3 (Remarks to the Author):

I thank the authors for their thoughtful addressing of my previous comments and concerns. I have no further comments.

Kostadin Kushlev

** Visit Nature Research's author and referees' website at www.nature.com/authors for information about policies, services and author benefits**

Open Access This Peer Review File is licensed under a Creative Commons Attribution 4.0 International License, which permits use, sharing, adaptation, distribution and reproduction in any medium or format, as long as you give appropriate credit to the original author(s) and the source, provide a link to the Creative Commons license, and indicate if changes were made.
In cases where reviewers are anonymous, credit should be given to 'Anonymous Referee' and the source.
The images or other third party material in this Peer Review File are included in the article's Creative Commons license, unless indicated otherwise in a credit line to the material. If material is not included in the article's Creative Commons

license and your intended use is not permitted by statutory regulation or exceeds the permitted use, you will need to obtain permission directly from the copyright holder.
To view a copy of this license, visit https://creativecommons.org/licenses/by/4.0/

January 12th, 2026

Dear Dr. Schubert:

We've attached our response to the reviews of our manuscript, "Affiliation in human-AI interactions based on shared psychological traits".

Here's a summary of the main changes:

- Reviewer 1 raised an important point on how adequate it was to assume that our main outcome variable was normally distributed and continuous. We acknowledged this possibility and explored how changing the primary analysis from a two step linear model to a non-parametric approach with Spearman correlations impacted the findings. This approach makes the result clearer whilst avoiding statistical assumptions and all results hold as in our original report (we report the parametric Linear Mixed Models in the Supplementary Information).

- Reviewer 2 requested an additional linguistic analysis to validate that the behaviour of the LLMs in the study was a reflection of their prompts (i.e., that the LLM mimicking anxiety actually produced anxious language). We tested this question by comparing our bot behaviour with a "bag-of-words" model, which captures the frequency of commonly used words; we also calculated weighted log odds ratios for frequently used words to capture a linguistic profile of each LLM. As we now report in the Results section, the language produced by the LLMs did match the prompted trait.

- Reviewer's 2 and 3 requested evidence that our adapted measure of affiliation was reliable, and valid as it relates to previously published measures of human-to-human affiliation. We had originally developed our questions from previous questionnaires but tweaked wording to reflect LLM affiliation for which there was no existing measure, but we recognise that these changes may have upset the integrity of the questions. We addressed this query in three ways: 1) we now demonstrate using Cronbach's alpha that our questionnaire is internally consistent (see Methods P. 6. and Supplementary Information "Affiliation Score"); 2) further, we used factor analysis to show that the questionnaire captures a single psychological factor; 3) we ran a control study (n=50) to compare responses on our questionnaire with a validated measure of human-to-human affiliation—the Connection During Conversations Scale (CDCS; Okabe-Miyamoto et al., 2024). The correlation between the two scales was highly significant [$r(49)=.89$, $p<.001$]. Taken together, this provides strong support for the hypothesis that our questionnaire measures a factor related to human-LLM affiliation.

In addition to these major changes, we've thoroughly edited the manuscript for concision and added text to the Discussion in response to requests made by Reviewer's 2 and 3.

We appreciate the opportunity to respond to our initial reviews; we feel that the paper is greatly improved and we hope that you and the Reviewers agree.

Sincerely yours,

Santiago Castiello, Riddhi Jain Pitliya, Daniel Lametti, and Robin Murphy

REVIEWER REPORTS
(LEGEND: **reviewer's comments**, our responses, new/edited manuscript text):

Reviewer #1 (Remarks to the Author):

It's a carefully done, important, timely study. It should be published. I just thought some issues could be made a little clearer earlier on in the manuscript. Specific comments below.

We thank the reviewer for their overall positive assessment of our manuscript.

Page 1, abstract. It might be helpful to define what it would mean for an LLM to exhibit the 'opposite' of someone's personality

We have added this information to the Abstract. The abstract now reads: "mimicked either their own personality profile or the inverse of their personality (i.e., the opposite pattern of Big-Five scores)."

Line 86 – examples of clinically relevant criteria here would be helpful rather than just directing the reader to a reference

Done.

P. 2: "In Experiment 1, the LLM GPT-4 was prompted using clinically relevant criteria—specifically, answers to the twenty-item State Trait Anxiety Inventory reflecting an anxious psychological state e.g., "I almost always feel nervous and restless" (Spielberger, et al. 1983)—to express through language a psychology aligned with either an anxious or non-anxious psychological state."

Line 89. Why was their anxiety measured after the conversation rather than before? Is there a chance that the conversation had a priming effect of some sort? Was it trait or state anxiety that was measured?

This is an important point and we thank the reviewer for raising it. We considered delivering the questionnaire before the conversations but we reasoned this might cue the participants to the aim of the interactions. Although it is possible that the conversations themselves primed responses, we note that every participant had a conversation with both the anxious and non-anxious (i.e., calm) LLMs (order counterbalanced). All things being equal, potential priming from one LLM would be cancelled out by the opposite prime from the other LLM. However it is also possible that those participants who received the anxious bot closer to the questionnaire may have been primed more closely to answering the questionnaire. To statistically examine this question we compared anxiety scores between participants who interacted with the anxious LLM first to participants who interacted with it last; If there was some differential priming then we might expect the scores to differ. In fact there was no difference in anxiety levels for the two orders [$t(87)=-.60$, $p=.549$].

Line 95. Again, what 'validated tests of personality' were used – more specifics/examples would be useful and appropriate.

Done.

P. 2: "GPT-4 was prompted using answers to twelve questions from the International Personality Item Pool (Goldberg, et al., 2006) to produce language aligned with either an extroverted or introverted personality."

Line 98. So the prediction was only about extroversion? Also, was this 'predicted' in advance as in a pre-registered hypothesis, or it's just informally what you expected might happen but you didn't pre-register that it would?

For Experiment 2, the prediction was that extroverted participants would affiliate more with the LLM that used extraverted language and vice versa for introverted participants. We did not preregister Experiments 1 and 2. Only Experiment 3 was preregistered.

Line 103. What is the 'inverse' of a person's personality profile?

We mean the opposite pattern of Big 5 personality scores (e.g., if the participant scored a 4/5 for extraversion the LLM would use language reflecting a score of 2/5). This has been clarified in the Introduction.

P. 2: "In a final preregistered experiment, we first measured participants' personality and then, for each participant, we created bespoke versions of the LLM (GPT 4.1) that mirrored the language aligned with either their exact personality profile or the inverse of their personality profile (i.e., an LLM that displayed the exact opposite pattern of Big 5 personality scores in the language it used)"

Line 124. Why did you settle on 100 to recruit?

As, at the time of testing, we were not aware of any existing studies examining human affiliation with LLMs during unstructured conversations, the sample size for Experiment 1 was estimated based on our pilot work and previous research that showed interactions between participant personality and chatbots using low hundreds of participants (e.g., Jin and Easton, 2023). Given that the effect in Experiment 1 was large (r=.34; r^2=.12; Cohen's d=.72; Std. Coef. = .34), we maintained this sample size for Experiments 2 and 3.

Line 125. Were the exclusion criteria registered?

The Exclusion criteria were not preregistered for Experiments 1 and 2 but they were for 3. As we explain below, the exclusion criteria were based on standard statistical techniques for outlier rejection.

Line 133. You recruited 100 b/c you anticipated exclusions?

For all experiments we anticipated some exclusions given the nature of online testing.

Line 151. Extra comma after "included"

Changed. Thanks for catching this.

Line 157. Maybe I missed it, but were participants explicitly told they'd be chatting with an AI? Or did they think they were chatting with a human? The introduction of Turing's test in the introduction might make it seem like this is a set up where they don't know but are

supposed to guess whether it's a human or an LLM they're conversing with. In any case, this key fact should be made clear in the beginning and be much more prominent.

Yes, participants were explicitly told they were chatting with an AI.
P. 2: The introduction reads: "The aim of the current experiments was to test whether, in a short unconstrained conversation, humans might affiliate more with an LLM that, through language, exhibits a shared psychology, despite full knowledge that they are talking to a computer program."

We have also added this information to the abstract: "With full knowledge that they were interacting with an artificial system".

Line 172. Why was 8 turns chosen as the exclusion criterion, and was this registered?

The exclusion criteria were defined using standard statistical techniques for outlier rejection—i.e., the mean number of conversational turns and the standard deviation of the sample. Fewer than 8 turns reflected conversations that were more than 2 SD away from the mean number of turns. From a conversational standpoint, eight turns was also the length of the LLM's context window in Experiments 1 and 2. We've added text to the Methods to clarify this.

P. 5: "In Experiments 1 and 2, each chat ended after 31 conversational turns or 10 minutes—whichever came first. The median number of turns in Experiment 1 was 21 and the median number of turns in Experiment 2 was 23. Participants who completed fewer than 8 turns—or more than 2 standard deviations away from the mean number of turns—were excluded. Fewer than eight turns was also selected as an exclusion criteria in these experiments because it was the length of the LLM's context window. In Experiment 3 participants were required to complete 24 conversational turns regardless of how long the conversations took."

For Experiment 3, we corrected this issue by strictly requiring 24 interactions. This has been clarified in the Methods section.

Line 184. First time I'm seeing the information that participants were told they were interacting with chatbots - this is really key information that needs to be highlighted earlier.

Thanks for flagging this. As noted above, we have now made this clear in both the Abstract and the Introduction.

Line 222 – something going on with the equation/formatting

Fixed.

Line 252. Is this assumption about Likert scales appropriate? Were the answers in fact normally distributed? Also, Likert scales are not continuous.

Thanks for raising this important point. You are correct that the approach we used in the original submission had some limitations. If we model the actual Likert values, the residuals are not normally distributed and Likert scales are not continuous. When we model the affiliation score, which is the average of 6 Likerts, it approximates a continuous variable. But the Linear Mixed Model is perhaps not ideal because each ID has only two observations. Thus, we changed the primary analysis strategy. Specifically, we used non-parametric Spearman correlations (we also present the parametric analysis using Linear Mixed Models as a Sensitivity Analysis in the

Supplementary Information, results are in the same direction). For Experiment 1 and 2 we took the affiliation score difference between each LLM personality and correlated that with the anxiety and personality scores to estimate the interaction, then individual correlations for each condition. For Experiment 3 we ran a non-parametric test as a sensitivity analysis in addition to the preregistered t-test. The results were consistent with what we previously reported.

We have added text to the Methods section (P. 6) to explain this new analysis which has no impact on the interpretation of the results..

Reviewer #2 (Remarks to the Author):

The work describes 3 empirical studies (one pre-registered) that GPT is perceived as eliciting greater affiliation when it matches the participant's personality. This is timely given to power and potential of LLMs to manipulate. The study is generally well-executed, but several methodological concerns must be addressed to make the results interpretable and convincing. The novelty appears limited given prior work on this topic, and the work needs to better position its novel contribution in light of prior work on social robots and agents. Some discussion of ethics seems necessary. The writing and design is clear with minor caveats.

NOVELTY: The author's claim that evidence for the similarity-attraction theory in agents has "little empirical support." However, there is extensive empirical evidence going back to work by Cliff Nass in the '90s and extending to the present day. This includes at least one meta-analysis on the topic. The authors do not cite or seem aware of this literature. While the paper may address limitations in this prior work, the novel contribution must be clarified. Some examples
- Moon and Nass 1996: Finds results consistent with similarity-attraction theory related to dominance.
- Nass and Lee 2001: showed similarity-attraction effects for extroversion
- Esterwood et al 2021: Mena-analysis of 13 similarity-matching studies in HRI

Note also the study used a single context (open-ended dialog) and a single measure of success (an unvalidated bespoke scale), lowering the novelty and generality of the results.

We thank the reviewer for sharing these papers related to attraction theory in relation to nonhuman agents. We have incorporated the above references into the introduction of our revision.

P. 1: "There is evidence that mimicry of a "psychology" in artificial systems might matter to humans. Meta-analytical evidence supports the idea that participants' personality matters for various forms of acceptance of robots[9]. In situations where people are able to identify dominant or submissive language in pre-programmed computer responses they feel more attracted to computers that are more similar to themselves in these traits[10]. A similar attraction effect is also found when people hear computer-synthesized speech[11]. And, more recently, extroverted participants were shown to deem scripted interactions with consumer-oriented chatbots as more enjoyable when the bot responded more quickly and used extroverted language and punctuation (e.g., "Hi there!" instead of "Hello")[12]. All the above studies broadly support the idea that affiliation, connection, and attraction can emerge during interactions with artificial agents. LLMs present a highly novel, AI-based tool to systematically test the limits of these effects."

It is important to note, however, that the papers cited above do not test the aim of the current study, which was to understand similarity-attraction in relation to human-LLM interactions. Although the suggested papers certainly provide a foundation for our work, we believe that interacting with an LLM—which can generate contextually correct human-like language on the fly—is significantly different than interacting with a chatbot or robot designed to deliver pre-programmed responses.

METHODOLOGY: There are a number of conceptual and methodological issues with the work that would need to be addressed before the work is suitable for publication. At minimum, these would require re-analysis and clarifying how these analyses relate to the pre-registered plan. It would likely require at least one new experiment.

Manipulation check: The authors prompt GPT to express a personality but the success of this prompting is never validated. Examples of validation would include recruiting a separate pool of human annotators or using pre-trained personality recognition models or linguistic analysis like LIWC. Though less convincing, this could include using another LLM to code dialogs.

Thanks for raising this important point. Although contemporary LLMs are exceptionally good at adopting a particular linguistic style, as you point out it's still important to verify that the manipulation was successful. We did this in two ways.

In the paper we analyzed the sentiment of messages sent by the LLM, which captures emotional tone (a component of the LIWC). The prediction in Experiment 1 was that an LLM displaying anxiety would send more negative sentiment messages and fewer positive sentiment messages than the LLM displaying a lack of anxiety. As shown in Figure 1B, this is exactly what happened. Similarly, in Experiment 2 the "extroverted" LLM sent more positive messages and fewer negative and neutral messages than the "introverted" LLM (Figure 2B). Thus, the sentiment or tone of the messages sent by the LLMs aligned with the personalities they were prompted to convey. We have edited the text in the Results to ensure that this important manipulation check is adequately conveyed.

Furthermore, we performed two additional lexical analyses. Specifically, we used a "bag-of-words" model to identify the top twelve words (by frequency of use) produced by each LLM in Experiments 1 and 2, removing filler words like "the". The results are shown below. The LLM prompted to mimic anxiety, used words like "hard", "feel" and "worry", while the non-anxious LLM used words like "enjoy", "fun", and "wonderful". For Experiment 2, the LLM prompted to mimic extroversion used words like "adventure", "fun", and "exciting", whereas the introverted LLM used words like "quiet", "peaceful", and "reading".

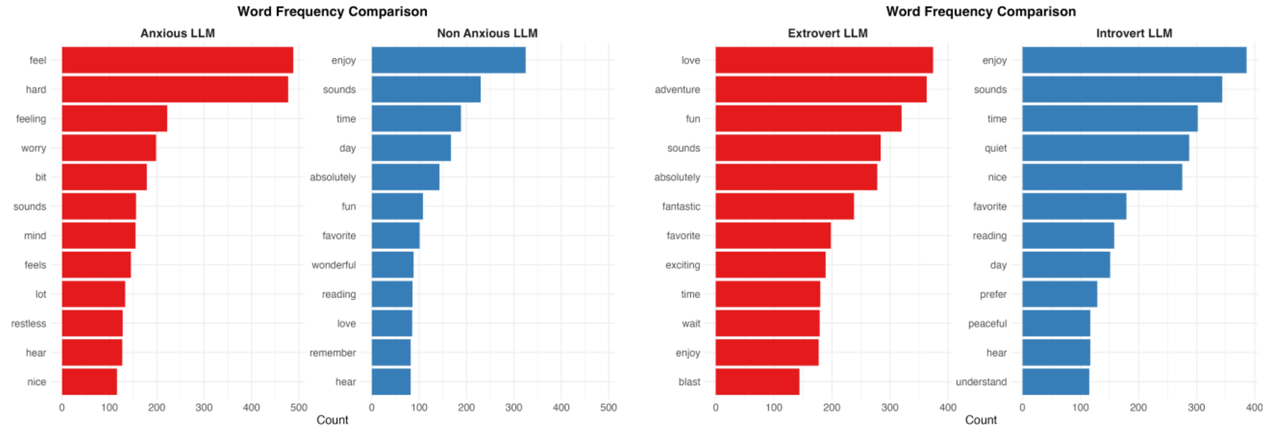

While raw word frequencies provide a descriptive overview, they do not capture the specific linguistic markers that distinguish one model's persona from another. To identify these markers, we calculated the weighted log-odds ratio between the two LLM personas for all words they produced in Studies 1 and 2 . This method identifies the distinctiveness of words by comparing their usage across conditions (anxious and nonanxious, for instance) while accounting for the total volume of text. The resulting scores are, effectively, z-scores: a value greater than 1.96 indicates that a word is significantly more characteristic of one persona than the other at $p < .05$.

The figure below shows the top 12 words with the highest weighted log odds ratio for the LLMs prompted to mimic anxiety and a lack of anxiety in study 1 (left panel), and the LLMs prompted to mimic extraversion and introversion in study 2 (right panel). The anxious LLM uniquely used words that signaled an anxious psychological state ("worry", "restless", "anxious"), whereas the non anxious LLM did not ("spirit", "exploring", "absolutely"). The extroverted LLM uniquely used words that signaled extraversion ("blast", "adventure", "climbing"), whereas the introverted LLM used words that aligned with introversion ("quite", "reading", "calm").

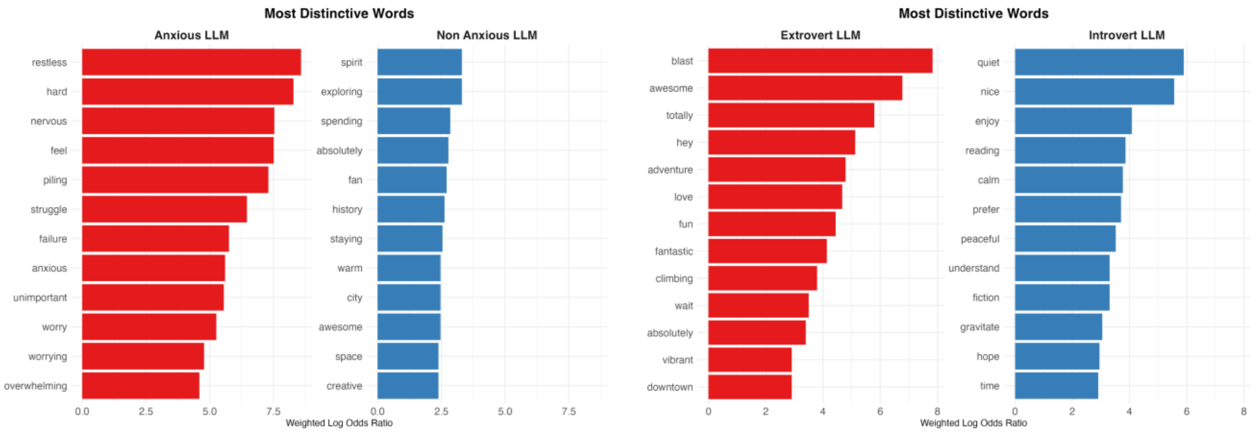

Thus, the language used by each LLM aligned with their assigned personalities.

We have added these figures to the Supplementary Information and altered the text in the Methods and Results sections to reflect this new linguistic analysis.

P. 7: "The sentiment of messages sent by both GPT-4 personas and participants was categorized as either Mixed, Negative, Neutral, or Positive (see Methods). This analysis had two aims: 1) to verify that an LLM instructed to mimic a negative emotional state (anxiety) produced more negative messages than an LLM instructed to mimic a positive emotional state (non-anxious); and 2) to test whether the sentiment of participants' messages was influenced by the sentiment of messages sent by the LLM in a manner that might relate to their self-reported affiliation with each LLM version.

As observed in Figure 2B (GPT-4 Texts), the two LLMs produced messages with markedly different sentiment profiles. To test this, we used a two-factor ANOVA [4 (sentiment) x 2 (LLM type)] and post-hoc paired t-test for each sentiment category. The interaction revealed differences in the GPT-4 personas between the sentiment categories ($F(704,3) = 498.64$, $p < .001$). When GPT-4 was instructed to mimic an anxious state it produced more negative ($t(88) = 14.88$, $p < .001$, $d = 1.58$ [1.26, 1.89]) messages and fewer positive ($t(88) = -27.67$, $p < .001$, $d = -2.93$ [-3.41, -2.45]) messages than when it was instructed to mimic a non-anxious state. To further verify that the LLMs used language that reflected their instructed personality, we identified words that were significantly more likely to be used by each LLM persona (as captured by a weighted log odds ratio, see Supplemental Data). When GPT-4 was instructed to mimic anxiety it uniquely used words like "worry", "anxious", and "nervous"; but when it was instructed to mimic a lack of anxiety it uniquely used words like "spirit", "absolutely", and "awesome" (see Supplementary Information). Taken together, this suggests that the LLMs mimicked the desired personality trait."

P. 11: "Similar to Experiment 1, the two GPT-4 personas produced messages with distinct sentiment patterns, reflecting the personality trait they were instructed to mimic (two-factor

ANOVA, interaction between sentiment and LLM type: $F(768,3) = 241.59$, $p < .001$). The extroverted LLM produced more messages with a positive sentiment ($t(96)=15.81$, $p<.001$, $d=1.61$ [1.30, 1.91]), fewer messages with a neutral sentiment ($t(96) = -16.01$, $p < .001$, $d = -1.63$ [-1.93, -1.32]), and less negative messages ($t(96) = -3.55$, $p < .001$, $d = -.36$ [-.56, -.15]) compared to the introverted LLM (Figure 3B, GPT-4 Texts). The extroverted LLM uniquely used words like "blast", "adventure", and "climbing" whereas the introverted LLM used words like "quite", "reading", and "calm" (see Supplementary Information)."

Taken together, we feel that these two analyses provide compelling evidence that the LLMs produced the instructed behaviour.

This is particularly important in explaining the failure to support the hypothesis when the trait is prompted to be low. For example, whereas we see the similarity-attraction effect when the model is prompted to be anxious or extroverted, we see no effect when the model is prompted to be non-anxious or introverted. The null findings in the low-trait conditions deserve more explicit discussion.

This is an interesting point. It is an empirical question as to whether different traits are equally expressed through language, and it might not be surprising that some traits are more easily expressed than others. Therefore, one explanation is that the smaller effects in the case where the trait is low relate to limitations in relation to expressing the trait through language. Humans might do a better job of recognizing intraversion, for example, based on observable actions. Anxiety, in contrast, is arguably a product of language (e.g., rumination).

We have added text to the Discussion to address this.

P. 15: "Interestingly, in Experiment 1 affiliation was not observed towards an LLM that displayed an absence of anxiety. Similarly, in Experiment 2, affiliation was not observed for the LLM that displayed introversion. One explanation for the lack of affiliation in these cases may relate to the challenge of distinctly conveying these traits through language. Whereas anxiety and worry is arguably enabled by language (e.g., rumination via internal speech; Borkovec and Inz 1990; Hirsch and Mathews 2012), and extraverted behaviours are easily described using language, it is likely more challenging to signal a calm or introverted state via a short conversation."

Problematic measure of attraction: the use of a "bespoke" measure is puzzling, given that there are many validated measures that could have been used. Given you innovate your own scale, you must report scale reliability (e.g, Cronbach's alpha). If the scale is not reliable, then you may wish to perform factor analysis, though your N is probably too low for this.

Thanks for raising this point. Although it's true that there are validated measures of similarity-attraction, there are no measures specific to human-LLM interactions, which is why we created a bespoke scale based on existing scales for humans. But as you rightly point out, it is important to validate our scale. We tackled this in three ways:

1) We computed Crombach's alpha for each experiment. The result—Expt. 1 = .91, Expt. 2 = .93, Expt. 3 = .88—indicated strong internal consistency for the six items in our questionnaire.

2) We ran exploratory factor analyses with the Minimum Residual method, and found that for all three experiments the best latent model contained a single factor. This suggests that our scale is measuring a single thing such as affiliation.

3) Finally, we ran a control study (n=50) in which participants had a single chat with GPT-4.1 instructed to be neutral in all Big 5 personality dimensions; we then asked participants to rate their experience using our 6-item bespoke rating scale and the validated 14-item Connection During Conversations Scale (CDCS) (Okabe-Miyamoto et al., 2024), order counterbalanced. Scores on our scale were highly positively correlated with the CDCS [$r(49)=.89$, $p<.001$].

Taken together, these additional analyses and data provide compelling evidence that our scale measured a single concept that was very similar to that measured by the Connection During Conversation Scale used to assess affiliation in human-to-human interactions.

We have incorporated this information into the Methods section and added the analyses to our Supplementary Information.

Reliance on within-subjects design: The use of a within-subjects design, and especially the choice to provide the scale only after interacting with both agents, will tend to amplify the effect and can lead to experimenter effects (e.g., when participants realize the goal of the study). This seems not ecologically valid as real users would not likely face such a stark choice in the real world. This should be discussed but a stronger paper would include a pre-registered Study 4 that uses a between-subjects design.

When designing the studies, we considered a between subjects design but, in the end, decided against this approach. Social interactions are often judged in relation to schemas constructed from past interactions (Baldwin, 1992). But human-LLM social interactions are a new phenomenon. Thus, our design—two counter-balanced LLM social interactions—gave participants a needed comparison to adequately judge their experience.

And while we agree that in studies 1 and 2 we presented participants with clear examples of the desired trait (e.g., anxiety vs a lack of anxiety). We are not convinced that such a comparison lacks ecological validity; many social situations involve stark comparisons between people (e.g., dating, working with new colleagues). We also note that in Experiment 3 the difference in personality between the two LLMs—i.e., the starkness of the choice—*depended* on how much each participant's own personality differed from the average. As we report, the size of the effect was related to this difference (Figure 3B). Thus, this key study both highlights and explains the reviewer's point—the starkness of the choice does matter, and thus affiliation with LLMs varies depending on the uniqueness of peoples' personalities in comparison to the history of exchanges they've had with LLMs.

Failure to analyze order effects: The use of a within-subjects design creates order effects. These should be analyzed and reported as they might change the interpretation of the results.

We analysed the effect of order on affiliation scores for experiments 1, 2, and 3 and we did not find any difference. We examined the impact of chat order on the affiliation scores using a linear ANOVA. No significant effects of order were found: Exp 1, beta = -.07, $p = .736$; Exp 2, beta = -.41, $p = .0502$; and Exp 3, beta = .14, $p = .498$. We included the complete analysis in the Supplementary Information and we reference it from the main text.

Incomplete reporting of results: The analysis should report the significance of the main effects and interaction terms (i.e., the sig of the coefficients corresponding to these factors). Also report effect sizes. Are these interesting effects?

Thanks for asking about this. We reported effect sizes in the form of standardized estimates. Given a suggestion from Reviewer 1, in this revision we used a non-parametric analysis with Spearman correlation tests for the main text (however effect sizes are reported in the Sensitivity Analysis where we used parametric statistics, see Supplementary Information). Now to test the interaction we report correlations between differences in conditions against questionnaire responses, thus no main effects were estimated along this. However to address this suggestion, we are also reporting affiliation differences between the conditions, which is equivalent to the main effect of the models. For example, P. 9: "Overall, participants affiliated more with the AI in the Nonanxious condition (M = .61) than the Anxious condition (M = .-20; t(88) = 5.86, p < .001, d = -.62 [-.8 to -.39]). And P. 11: "Overall, participants' affiliation did not differ between the Extrovert (M = .55) and the Introvert (M = .36) conditions (t(96) = 1.43, p = .157, d = .14 [-.06 to.34])."

Other issues:
- The nature of the task was hard to discern from the main text. It would help to have the exact instructions. Given the description, and that participants make the first dialog move, it seems possible there is considerable variance in the conversations

The actual instructions were added to the Supplementary Information. Participants were instructed to get to know the LLM as if they were texting with a new friend. In all the cases the LLMs started the conversation, and this has been clarified in the methods: "The AI always started the conversations."

By design, the task involved unstructured conversations with the LLMs. Given the nature of the task, there was significant variance in the conversations. This approach, although unconstrained, has the highest ecological validity.

- Objective analysis of the text might help unpack causality as to who is influencing who. Right now, some results are described as causal (e.g., participant adapts to agent) when they are simply correlational (e.g., discussion of fig 2b&c)

We have edited the text to tone down language that implies causality where it is not warranted.

- The description of how the experiment was implemented is hard to follow and seems to assume some familiarity with GSX. The way that long dialog contexts are handled is particularly unclear (Exp 1&2).

Thanks for flagging this. We have updated the methods section to clarify this. In our pilot work, we found that GPT-4 sometimes struggled to maintain the prompted personality over the length of a long conversation. To solve this problem, we restricted its context window to the prompt and the most recent eight conversational turns. Thus, its responses were always a strong reflection of the prompt and the most recent exchanges with participants. By the time we ran Experiment 3, GPT4.1 had been released and its ability to follow instruction over long conversations had significantly improved and thus we did not limit the LLM's context window. The text in the Methods now reads P. 4: "In Experiments 1 and 2 the LLM's context window was restricted to eight conversational turns. At this point, the first two turns were ejected after every subsequent turn so that the context

window never grew beyond the prompt, example responses, and the most recent eight turns. Limiting the LLM’s memory to the prompt and the last eight turns ensured that the model’s persona was always a strong reflection of the prompt and the most recent exchanges with participants, and did not drift towards language used by the participant.”

- I would suggest against calling out specific BFI items in the description of Study 3: this goes in supplemental. Just describe conceptually.

Done. We moved the individual items to a small section in Supplementary Information called “Big-Five 44 items”.

- Focus on the uncanny valley seems odd and distracts from your point. You never assess if the dialog seems human-like or uncanny. Your presumption is they are all human-like but you are simply manipulating style (note there is plenty of literature suggesting that LLMs have a default personality – e.g., agreeable) so it is not like you are explicitly trying to make them more/less human-like.

We appreciate this point. Although the analogy isn’t perfect, we feel that this literature does provide a foundation for this work. Although it’s true that LLMs do have a default personality, this personality is overly agreeable and always lacks characteristics with a negative valence that (arguably) define human personalities (e.g., anxiety, neuroticism, disagreeableness). So, we do feel that we are effectively giving LLMs more human-like personality traits. Given the artificial nature of the interaction (which was clear to participants) this presented the possibility of a negative (or valley-like) reaction.

In sum, this work raises interesting questions about personality matching in LLM-mediated interaction, but its methodological and theoretical framing require substantial revision before it can make a reliable contribution to the literature

We appreciated the overall positive sentiment and constructive feedback; we believe that the changes we have made clarify your questions.

Reviewer #3 (Remarks to the Author):

This is a review of the manuscript entitled "Affiliation in human-AI interactions based on shared psychological traits." The paper is timely, methodologically solid, and relatively well-written. A key strength of the paper is the fact that the authors take a well-established psychological phenomenon (similarity leads to affiliation and liking) and examine whether this phenomenon applies when people chat with an LLM-powered chatbot. The paper's findings thus have theoretically-grounded implications for understanding human-AI interactions and relationships, as well as practical applications for designing AI that is better able to facilitate rapport between humans and AI agents. Previous research does suggest that mimicry, when too obvious, can backfire, so the findings of the paper, while not surprising, are far from obvious either. Overall, this paper is a good example of how new phenomena, like LLM-powered chatbots, can be rigorously studied and connected to established psychological theories and findings.

We thank the reviewer for this positive assessment of our manuscript.

All of that said, I do have one concern that I would classify as major. Specifically, the key outcome measure is an ad-hoc, or bespoke, measure of six items. While face-valid, this measure is not validated or used in previous research. Thus, it is not clear how the findings of this paper really relate to the more established findings around similarity, liking, and affiliation. This is not a minor issue because ultimately, without grounding and validation, it is not clear what we are measuring, whether or not we can call it affiliation, or how it relates to other constructs. For example, in the Discussion, the authors seem to be interpreting the finding as an indicator of a sense of connection that humans experience with the AI ("…through language could foster a sense of connection between humans and AI."). But there are established measures of liking and sense of connection that are not employed here, so we do not know if this is the correct interpretation of the findings.

I can see several ways that this issue can be resolved. First, the authors could run a "pilot study" where they validate this measure against a range of established measures of connection, liking, and affiliation. This will make the existing studies more convincing. Second, the authors could re-run one of the studies (e.g., Study 3) with more measures that are established and validated. At the very least, though, this issue needs to be addressed head-on in the Discussion.
Relatedly, the discussion is very sparse on acknowledging limitations of the study (e.g., MTurk sample, findings that did not confirm hypotheses, use of unvalidated measures, and so on).

Thanks for raising this issue. Reviewer 2 had similar concerns, and so we've taken a number of steps to reassure readers that our bespoke questionnaire is valid.

1) We computed Crombach's alpha for each experiment: Experiment 1 = .91, Experiment. 2 = .93, and Experiment 3 = .88. This suggests that the items in the questionnaire are correlated and measure the same underlying trait.

2) We ran factor analyses with the Minimum Residual method, and found that for all three experiments the best latent model contained a single factor. This suggests that our scale is measuring a single thing i.e., affiliation/liking.

3) Finally, we ran a control study (n=50) in which participants had a single chat with GPT-4.1 instructed to be neutral in all Big 5 personality dimensions; we then asked participants to rate their experience using our 6-item bespoke rating scale and the validated 14-item Connection During Conversations Scale (CDCS, Okabe-Miyamoto et al. 2024), order counterbalanced. Scores on our scale were positively correlated with the CDCS ($r(49)=.89$, $p<.001$).

Taken together, these additional analyses and data provide evidence that our scale measured a single concept that was very similar to that measured by the Connection During Conversation Scale used to assess human-to-human interactions.

We have incorporated this information into the Methods section and added the analyses to our Supplementary Information.

The decision to exclude certain participants in Studies 1 and 2 without that decision or criteria being preregistered warrants more explanation. How were the exclusion criteria derived? What measures were taken to ensure that the criteria were not influenced by the results of the study (e.g., were they set before looking at the data)? I would prefer seeing the results without excluding participants, at least in SOM.

The exclusion criteria were defined using standard statistical techniques for outlier rejection—i.e., the mean number of conversational turns and the standard deviation of the sample. Fewer than 8 turns reflected conversations that were less than 2 SD below the mean number of turns. From a conversational standpoint, eight turns was also the length of the LLM's context window in Experiments 1 and 2. We've added text to the Methods to clarify this, p. 5: "In Experiments 1 and 2, each chat ended after 31 conversational turns or 10 minutes—whichever came first. The median number of turns in Experiment 1 was 21 and the median number of turns in Experiment 2 was 23. Participants who completed fewer than 8 turns—or less than 2 standard deviations below the mean number of turns—were excluded. Fewer than eight turns was also selected as an exclusion criteria in these experiments because it was the length of the LLM's context window. In Experiment 3 participants were required to complete 24 conversational turns regardless of how long the conversations took."

There are some typos throughout the manuscript.

The writing is generally clear. However, the second paragraph, where the Turing test is talked about, is not well integrated into the narrative, and it is not clear how the Turing test has anything to do with the subject matter of the paper (or at least why it deserves an entire paragraph). Some sentences are unclear and need revision. For example, I have a hard time understanding what the following sentence means: "But the degree to which people feel more similar to an AI that reflects characteristics of their own psychology remains unknown." Finally, the Methods section was quite difficult to understand because statistical tests are described without explaining what question each statistical test would answer. This becomes a bit clearer in the Results, but it needs to be clearer why certain tests are being performed and how they build on each other to address the RQs.

We appreciated this feedback. We have thoroughly edited this revision to fix these problems. We have also removed the paragraph that discusses the relevance of the Turing test.

Finally, in Studies 1 and 2, the hypotheses seem only partially supported. Specifically, people's own traits are not related to how affiliated they feel with the non-anxious and

introverted chatbots. Why is that? As far as I can tell, this finding is not predicted. Is there a reason the traits are only related to affiliation for the anxious and extroverted bots? More discussion is needed on this point, and it needs to be acknowledged as a limitation (since this pattern was not predicted and appears inconsistent with the hypothesis).

Reviewer 2 was also interested in this aspect of the results. We've added text to the Discussion to address this. In short, we feel like "calmness" and "introversion" may be hard traits to convey in a short conversation because they reflect a *lack* of a signal, whereas anxiety and extraversion can be clearly signaled using language.

P. 15: "Interestingly, in Experiment 1 affiliation was not observed towards an LLM that displayed an absence of anxiety. Similarly, in Experiment 2, affiliation was not observed for the LLM that displayed introversion. One explanation for the lack of affiliation in these cases may relate to the challenge of distinctly conveying these traits through language. Whereas anxiety and worry is arguably enabled by language (e.g., rumination via internal speech, (Borkovec and Inz 1990; Hirsch and Mathews 2012)), and extraverted behaviours are easily described using language, it is likely more challenging to signal a calm or introverted state via a short conversation."

I hope my review is helpful.

We greatly appreciate the feedback; we feel like the manuscript is significantly improved.

References:

Baldwin, M. W. (1992). Relational schemas and the processing of social information. *Psychological Bulletin*, *112*(3), 461–484.

Okabe-Miyamoto, K., Walsh, L. C., Ozer, D. J., & Lyubomirsky, S. (2024). Measuring the experience of social connection within specific social interactions: The Connection During Conversations Scale (CDCS). *PloS One*, *19*(1), e0286408.
